# The role of naturally occurring and vaccine-induced Ad26 neutralizing antibodies in immunogenicity of Ad26-based vaccines

**DOI:** 10.1038/s41541-026-01488-8

**Published:** 2026-05-21

**Authors:** Mariska G. M. van Rosmalen, Joris Menten, Daniel J. Stieh, Nadine Salisch, Janine van Duijn, Arangassery Rosemary Bastian, Chelsea McLean, Viki Bockstal, Kim Stuyckens, Macaya Douoguih, An Vandebosch, Hanneke Schuitemaker, Jenny Hendriks

**Affiliations:** 1 Johnson & Johnson, Leiden, The Netherlands; 2 Johnson & Johnson, Beerse, Belgium

**Keywords:** Diseases, Immunology, Medical research, Microbiology

## Abstract

Adenovirus 26 (Ad26) is frequently used as a vaccine vector targeting infectious diseases. Neutralizing antibodies (NAbs) induced by natural infection or immunization against some viral vaccine vectors may impact vaccine-induced immunogenicity and efficacy. We evaluated the natural seroprevalence and impact of Ad26 NAbs on vaccine immunogenicity using data combined from 21 clinical studies including 3851 participants from 15 countries. Ad26 NAb seroprevalence was higher in Africa than the United States and Europe. An inconsistently observed, clinically negligible negative trend was observed between naturally occurring Ad26 NAbs and vaccine-induced immune responses in participants who received Ad26-based Ebola or HIV vaccine regimens. Assessment of the impact of vaccine-induced anti-vector immunity after repeated vaccination found that an Ad26-based COVID-19 vaccine received 1–12 months before an Ad26-based RSV vaccine did not seem to impact post vaccination RSV immune responses. Within the study limitations, our data suggest that Ad26-based vaccines can be used in countries with high Ad26 seroprevalence and for repeated vaccination without compromising the vaccine antigen-induced immune response.

## Introduction

Adenoviral vectors have been used as a platform for vaccines approved for use for the prevention of coronavirus-19 disease COVID-19, and Ebola, and have been investigated for other indications including respiratory syncytial virus (RSV) human immunodeficiency virus (HIV), influenza, rabies, malaria, Zika and human papillomavirus^1–6^. However, anti-vector immunity can be a potential limitation of this platform, leading to reduced immune responses against the target antigen. Immunity against viral vectors develops due to previous natural exposure to the virus from which the vector is derived (naturally occurring), or due to previous vaccination with a viral vector-based vaccine (vaccine induced). The seroprevalence and magnitude of neutralizing titers following naturally occurring immunity vary by adenovirus serotype, geographic location, and to a lesser degree, age and sex^7–11^.

The impact of anti-vector immunity on vaccine responses has been extensively studied for vaccines based on Adenovirus 5 (Ad5). Two studies assessing an Ad5-based HIV vaccine showed that increasing titers of Ad5 neutralizing antibodies (NAbs) were associated with a decrease in interferon-gamma (IFN-γ) secreting HIV-specific T cells^12–14^. Studies of an Ad5-based Ebola vaccine with homologous booster and an Ad5-based COVID-19 vaccine (with or without homologous booster) showed that participants with pre-existing Ad5 NAbs titers >1:200 had considerably lower vaccine insert–specific binding and NAbs post-vaccination than those without high levels of pre-existing immunity^15–17^. T-cell responses (independent of type-specific NAbs) and innate immune responses against the Ad5 vector were also found to affect Ad5-based vaccine responses to the target antigen^18,19^.

Differences in the degree of impact that anti-vector immunity has on immune responses to the target antigen have been described between different adenovirus subtypes and disease targets, and the results have not always been consistent. For a chimpanzee adenovirus (ChAd)Ox1-based COVID-19 vaccine, some studies reported no relationship^20–22^ while another reported a minor negative correlation^23,24^. For a ChAd3-based Ebola vaccine, no correlation between pre-existing vector antibodies and humoral or CD4 T-cell responses was found, but a moderate negative correlation with the CD8 T-cell response was observed^25^. Adenovirus subtypes that are associated with infections in different species can differ with respect to cell-entry receptor usage, target-cell and tissue tropism, uncoating, endosomal escape, and interaction with innate immune sensors^26,27^. To what extent these different characteristics are associated with an adenoviral vector’s sensitivity to anti-vector immunity is unknown.

Ad26 (a serotype of *Human mastadenovirus D)* has been recognized as a promising vector for vaccine development^6^ and the seroprevalence of naturally occurring anti-vector NAbs is generally lower for Ad26 compared to Ad5^7,8^. Individual randomized trials with Ad26-based vaccines targeting COVID-19^28–33^, Ebola^1,34–38^, HIV^39–46^, RSV^47–49^, and Zika^50^, conducted in different countries throughout the world, have not reported an impact of either naturally occurring or vaccine-induced NAbs against the Ad26 vector following individual doses or multi-dose regimens. Of note, repeated administration of Ad26-based vaccines with the same insert showed acceptable vaccine immunogenicity despite the presence of vaccine-induced vector immunity^29–31,43–45,49,50^, and booster responses were observed^33,38,51–53^.

To date, there has been no global evaluation of the impact of pre-existing Ad26 immunity on responses to Ad26-vectored vaccines. We investigated the potential impact of Ad26 NAbs on the immunogenicity of Ad26-based vaccines by conducting a pooled analysis of data to evaluate the seroprevalence and titers of Ad26 NAbs within different study populations across various geographic regions, as well as the impact of these naturally occurring Ad26 NAb titers on the immunogenicity of two Ad26-based vaccines (HIV and Ebola). Furthermore, Ad26-based vaccine-induced immunogenicity (RSV) was assessed after repeated vaccination with two heterologous Ad26-based vaccines (COVID-19 followed by RSV) to determine whether vaccine immunogenicity was hampered in populations with a high prevalence of vaccine-induced Ad26 immunity.

## Methods

### Study data

This study used blood samples collected from participants in clinical trials of Ad26-vector Ebola, HIV, and RSV vaccines investigated by Johnson & Johnson. The list of studies included in the analysis is provided in Table 1. The individual studies have been reported separately. All protocols were approved by appropriately convened ethics review boards and informed consent was obtained from all participants, as detailed in the individual publications/reports.

**Table 1:** Overview of studies included in the seroprevalence, immunogenicity, and repeat adeno datasets

Vaccine program	Study no.	Vaccine regimen	No. of participants in datasets		
			Prevalence	Ebola and HIV immunogenicity	Repeat adeno
Ebola	VAC52150EBL1001	Ad26.ZEBOV, MVA-BN-Filo	51	14	-
	VAC52150EBL1002	Ad26.ZEBOV, MVA-BN-Filo	63	-	-
	VAC52150EBL1003	Ad26.ZEBOV, MVA-BN-Filo	36	15	-
	VAC52150EBL1004	Ad26.ZEBOV, MVA-BN-Filo	35	15	-
	VAC52150EBL2002	Ad26.ZEBOV, MVA-BN-Filo	501	99	-
	VAC52150EBL2003	Ad26.ZEBOV, MVA-BN-Filo	176	-	-
	VAC52150EBL3001	Ad26.ZEBOV, MVA-BN-Filo	880	241	-
	VAC69120FLV1001	Ad26.Filo, Ad26.ZEBOV	65	11	-
Subtotal			1807	395	-
HIV	HIV-V-A004	Ad26.Mos.HIV	390	-	-
	VAC89220HPX1002	Ad26.Mos.HIV w/o protein	36	-	-
	VAC89220HPX2003	Ad26.Mos4.HIV w/o protein	152	101	-
	VAC89220HPX2004	Ad26.Mos.HIV, Ad26.Mos4.HIV w/o protein	198	93	-
Subtotal			776	194	-
RSV	VAC18192RSV1001	Ad35.RSV.FA2, Ad26.RSV.FA2	44	-	-
	VAC18192RSV1003	Ad35.RSV.FA2, Ad26.RSV.FA2	31	-	-
	VAC18193RSV1003	Ad26.RSV.preF	72	-	-
	VAC18193RSV1004	Ad26.RSV.preF Ad26.RSV.preF/protein	626	-	-
	VAC18193RSV1005	Ad26.RSV.preF	24	-	-
	VAC18193RSV2001	Ad26.RSV.preF/protein	194	-	-
	VAC18193RSV2002	Ad26.RSV.preF	61	-	-
	VAC18193RSV2003	Ad26.RSV.preF	178	-	-
	VAC18194RSV2001	Ad26.RSV.preF	38	-	-
	VAC18193RSV3001	Ad26.RSV.preF/protein	-	-	348 exposed, 441 unexposed
Subtotal			1268	-	
Grand Total			3851		348 exposed, 441 unexposed

Four datasets were generated for analysis. The study cohorts included all participants with a non-missing Ad26 viral neutralizing assay (VNA) value at baseline and who were part of the per-protocol immunogenicity (PPI) set as defined in each individual study following rules defined in the study-specific statistical analysis plans.

To study seroprevalence and titers of naturally occurring Ad26 NAbs, a seroprevalence set was created containing pooled pre-vaccination (active or placebo) Ad26 VNA data (*n* = 3851) from locked and released databases of clinical studies conducted as part of three Ad26-based vaccine programs up to January 2020: Ebola (eight studies), HIV (four studies), and RSV (nine studies) (Table 1). Data from all age groups were included.

To study the impact of naturally occurring Ad26 NAbs on the immunogenicity of two Ad26-based vaccines, two subsets were created including data from adults aged 17–60 years who received a specific vaccination regimen. The Ebola immunogenicity set comprised data from participants who had received Ad26.ZEBOV (5 × 10^10^ virus particles [vp]/dose), a monovalent, recombinant, replication-incompetent Ad26 vector vaccine encoding the glycoprotein of the Ebola virus (Mayinga variant), followed by a non-replicating, recombinant, modified vaccinia Ankara (MVA) vector-based MVA-BN-Filo vaccine encoding glycoproteins from Ebola virus (Mayinga variant), Sudan virus (Gulu variant), and Marburg virus (Muscow variant), and nucleoprotein from the Taï Forest virus (1 × 10^8^ infectious units/dose), given at a 56-day interval. This Ebola immunogenicity set contained pooled pre- and post-vaccination Ad26 VNA, Ebola virus glycoprotein (EBOV GP) Filovirus Animal Nonclinical Group (FANG) enzyme-linked immunosorbent assay (ELISA), EBOV GP pseudovirion VNA (psVNA), and IFN-γ enzyme-linked immunospot (ELISpot) data from 395 adults who participated in six studies.

**Fig. 1:**
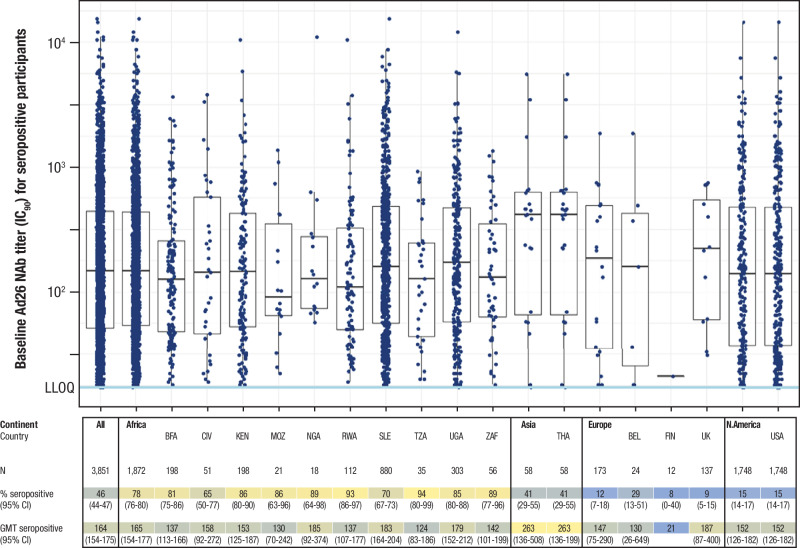
Naturally occurring Ad26 NAb titers (IC_90_) at baseline by continent and country. A boxplot showing the median (50th percentile) Ad26 NAb titers of Ad26 seropositive participants with boxes representing 25th percentile to 75th percentile. Whiskers extend to the lowest and highest value that is within 1.5 times the interquartile range. The individual participants’ datapoints are plotted over the boxplot as jittered and colored dots. The table provides an overview of the total participants (N) and the % Ad26 seropositive participants and their respective GMT per continent and country. The % seropositive and GMTs are color coded with the lowest value being blue and the highest value being yellow. Note. Asia is exclusively Thailand and N. America is exclusively United States. BEL Belgium, BFA Burkina Faso, CIV Côte d’Ivoire, FIN Finland, GMT geometric mean titer, KEN Kenya, LLOQ lower limit of quantitation, MOZ Mozambique, NAb neutralizing antibody, NGA Nigeria, N. America North America, RWA Rwanda, SLE Sierra Leone, THA Thailand, TZA Tanzania, UGA Uganda, UK United Kingdom, USA United States of America, ZAF South Africa.

The HIV immunogenicity subset assessed Ad26.Mos4.HIV, a tetravalent vaccine composed of four recombinant, replication-incompetent Ad26 vectors encoding mosaic Gag and Pol or Env proteins of HIV-1. The following four active pharmaceutical ingredients were pre-mixed in a 1:1:1:1 vp ratio: Ad26.Mos1.Gag-Pol (recombinant, replication-incompetent Ad26 encoding Mosaic 1 [Mos1] HIV-1 Gag and Pol proteins); Ad26.Mos2.Gag-Pol (recombinant, replication-incompetent Ad26 encoding Mosaic 2 [Mos2] HIV-1 Gag and Pol proteins); Ad26.Mos1.Env (recombinant, replication-incompetent Ad26 encoding Mos1 HIV-1 Env protein); Ad26.Mos2S.Env (recombinant, replication-incompetent Ad26 encoding modified Mos2 HIV-1 Env protein). Immunization schedules consisted of a total of four vaccinations: Ad26.Mos4.HIV was administered at Day 0 and Week 12 in participants who received the two active regimens; at Weeks 24 and 48 participants were subsequently administered contralateral injections of Ad26.Mos4.HIV and either clade C glycoprotein 140 (gp140) plus adjuvant or clade C gp140 and Mosaic gp140 plus adjuvant. This HIV immunogenicity set contained pooled pre- and post-vaccination Ad26 VNA, HIV-1 Env-specific ELISA and VNA, and IFN-γ ELISpot after stimulation with HIV-1 peptides pools containing potential T-cell epitopes of Env, Gag, and Pol data from 194 adults who participated in two studies. For both immunogenicity sets, data were split by geographic region based on Ad26 seroprevalence in Africa (high) versus the United States and Europe (low, Fig. 1).

To study the impact of previous Ad26-based vaccination on subsequent Ad26-based vaccine-induced immunogenicity, a repeat adeno set was created including 348 participants aged ≥60 years who received ≥1 dose of COVID-19 vaccine (Ad26.COV2.S [5 × 10^10^ vp/dose) prior to an RSV vaccine (Ad26.RSV.preF [1 × 10^11^ vp/dose] in combination with RSV preF protein [150 µg]) (exposed subset) and 441 participants aged ≥60 years who did not receive any adenovirus-vectored vaccine prior to the RSV vaccination (nonexposed control subset, representative of the overall study population but not matched to the exposed subset). Data for the repeat adeno analysis was from the unblinded dataset of the primary analysis of a single RSV study (VAC18193RSV3001; NCT04908683) at the time of database lock of October 4, 2022. For the exposed subset, all participants receiving an RSV vaccination in the PPI population in the United States who had previously received at least one COVID-19 vaccination (Ad26.COV2.S) between 12 months to 28 days prior to RSV vaccination were included. For the unexposed control subset, a sample of participants in the PPI population in the United States who received an RSV vaccination and who had no record of prior exposure to any adenovirus vectored COVID-19 vaccine were included.

Unexposed participants preselected in the immunogenicity subset and as controls in a separate correlates-of-protection analysis were selected for the control subset. Additionally, review of the available data was performed after unblinding, but before start of the analysis (i.e., before release of RSV preF IgG level data for the exposed subset), to assess the size of the control group and the amount of overlap in baseline characteristics between the exposed and the unexposed controls groups. It was observed that the number of selected unexposed controls would potentially be lower than the number of exposed individuals, which might limit the power of the analysis. To ensure an unexposed:exposed ratio >1:1, an additional set of 150 unexposed controls were randomly selected.

The repeat adeno dataset contained pre-vaccination Ad26 VNA, and Day 15 post-vaccination RSV subtype A stabilized prefusion protein (PreF A) IgG levels and RSV-A2 VNA titers.

For a more detailed overview of the study data in these subsets see Table 1 and Supplementary Table S1. Details on assay descriptions can be found in the individual study publications provided in Supplementary Table S1.

### Ad26 NAbs

Baseline Ad26 NAb seropositivity and geometric mean titers (GMTs) were evaluated with a type-specific VNA at two different locations; details of the assay have been previously described^54^. In the Leiden assay, titers ≥lower limit of quantitation (LLOQ; IC_90_ of 16 for manual assay or 17 for automated assay) were considered positive and titers <LLOQ were negative (imputed to 17). The LLOQ of the Nexelis assay was 21 IC_90_ which would underestimate seropositivity with respect to the Leiden assay. For consistency in titer levels, the lower limit of detection (LLOD; IC_90_ of 16) was used as cutoff for positivity in the Nexelis assay (used only for analysis of repeat adeno set). Thus, in the Nexelis assay titers ≥LLOD were considered positive, titers <LLOD were negative (<LLOD were imputed to LLOD/2). For the Nexelis assay, titers between LLOD and LLOQ were imputed to LLOD + [[LLOQ − LLOD]/2]). During the official transfer of the assay, it was demonstrated that the assay generated equivalent results and all concordance criteria were met.

Seroconversion was defined as being negative (Ad26 VNA titer Leiden <LLOQ or Nexelis < LLOD) at baseline and positive (Leiden ≥ LLOQ or Nexelis ≥ LLOD) following vaccination. The magnitude of positive Ad26 VNA titers was arbitrarily demarcated into three ranges: ≤200 (low), >200 to ≤1000 (moderate), and >1000 (high)^7,8^.

### Insert-specific responses in Ebola and HIV immunogenicity sets

Because sample collection timepoints across the clinical studies varied, two timepoints of interest were selected for analysis of the impact of pre-existing Ad26 NAbs. Participant individual peak responses were defined as the maximum observed titer/concentration obtained <4 months (120 days) after the last vaccine administration. Durability of the response was defined as the last titer/concentration obtained ≥4 months after the last vaccine administration. The 4 month cut-off for discriminating between peak and durability was based on the observed decline in immunogenicity response post vaccination and availability of timepoints with immunogenicity assessment (individual vaccine responses over time are provided in Supplementary Fig. S1) and supported by Alexandre et al. and Dari et al.^55,56^. When there was >1 durability result for a participant, the latest measurement was included in the analysis.

### Statistical analysis

The analysis of the seroprevalence and immunogenicity datasets was exploratory and based on frequency tabulations and graphical displays. No hypothesis testing was performed.

In the repeat adeno analysis, the impact of prior vaccination with a COVID-19 vaccine (Ad26.COV2.S) on the immunogenicity of the RSV vaccine (Ad26.RSV.preF/protein) was estimated as the GMT ratio and 95% confidence interval (CI) of the RSV preF IgG levels and RSV-A2 VNA titers with and without prior COVID-19 vaccination. The GMT ratio was estimated by comparing the Day 15 RSV preF IgG levels and RSV-A2 VNA titers between study participants in the exposed group and unexposed control group. Inverse probability weighting was used to adjust for differences in the distribution of baseline predictors of the immune response between participants with and without prior COVID-19 vaccination (Fig. S2). The inverse probability weighting analysis was performed using machine learning techniques (ensemble modeling) with propensity scores obtained by combining eight candidate models with up to 10 potential confounding variables using the SuperLearner package in R version 4.1.3. Confounders included in the models were age, sex, body mass index, ethnicity, region, smoking status, baseline preF A IgG levels, baseline RSV-A2 VNA titers, and high risk of RSV as defined by the US Centers for Disease Control and Prevention at the time of the study – narrow and broad definitions, see (Fig. S2). HIV status or a history of hepatitis or liver disease were included in the pre-defined list of possible confounders but were not included in the model as there were fewer than 10 participants in either the exposed or non-exposed analysis groups.

## Results

### Prevalence of naturally occurring Ad26 NAbs

The prevalence of Ad26 NAbs varied by continent, with the highest seroprevalence reported in Africa (78%), followed by Asia (41%, exclusively Thailand), and comparatively low seroprevalence levels in North America (15%, exclusively United States) and Europe (12%; Fig. 1). Within Africa, seroprevalence varied substantially by country, ranging from 65% in Côte d’Ivoire to 94% in Tanzania. Within participants seropositive for Ad26 NAbs, GMTs and medians were consistent between continents and countries (Fig. 1). The overall GMT in Ad26-seropositive participants was 164 IC_90_.

Prevalence and titers were analyzed with respect to sex, body mass index (BMI), and age (Supplementary Figs. S3, S4, and S5). Seroprevalence and absolute titers did not appear to differ between males and females. seroprevalence, but not titers, appeared slightly increased in higher BMI categories based on observations in the United States. seroprevalence increased with increasing age, with a substantial increase observed from childhood to early adolescence in Africa. seroprevalence in country-level data that included data from children (Burkina Faso, Uganda, Côte D’Ivoire, Kenya, Sierra Leone, and Finland) might therefore be an underestimation of seroprevalence in the adult population.

### Association between naturally occurring Ad26 NAbs and Ebola vaccine-induced responses

**Fig. 2:**
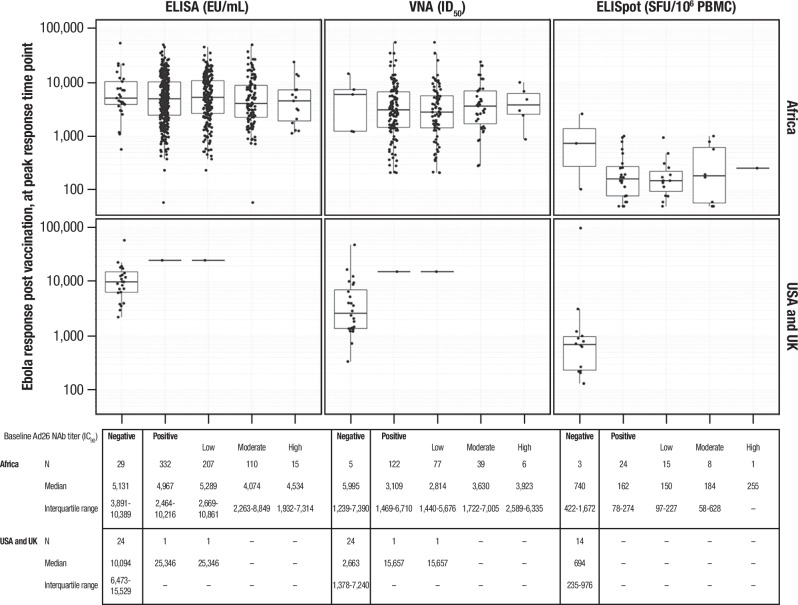
Vaccine-induced peak immune responses post Ebola vaccine regimen. Boxplots presenting the median (50th percentile) Ebola response measured by ELISA, VNA, and ELISpot post vaccination (Ad26.ZEBOV followed by MVA-BN-Filo at 56-day interval) at the peak timepoint (maximum observed titer/concentration observed <4 months post last vaccination) for participants from Africa versus the United States and United Kingdom with boxes representing 25th percentile to 75th percentile. Whiskers extend to the lowest and highest value that is within 1.5 times the interquartile range. The individual participant’s datapoints are plotted over the boxplot as jittered dots. The corresponding table provides an overview of the total participants (N) and the median response and interquartile ranges. Participants were seronegative if the Ad26 NAb titer (IC_90_) was <LLOQ and seropositive if the Ad26 NAb titer (IC_90_) was ≥LLOQ. The magnitude of positive Ad26 NAb titers was demarcated into 3 ranges: ≤200 (low), >200 to ≤1000 (moderate), and >1000 (high). African countries included Burkina Faso, Côte d’Ivoire, Kenya, Sierra Leone, Tanzania, and Uganda. ELISA enzyme-linked immunosorbent assay, ELISpot enzyme-linked immunospot, LLOQ lower limit of quantitation, NAb neutralizing antibody, PBMC peripheral blood mononuclear cell, SFU spot forming units, VNA virus neutralization assay.

**Fig. 3:**
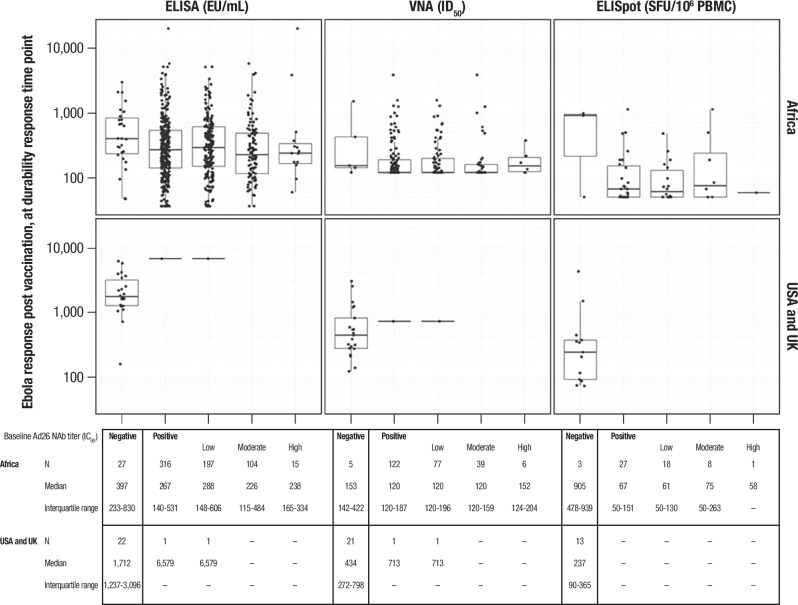
Vaccine-induced durability of the immune responses after an Ebola vaccine regimen. Boxplots presenting the median (50th percentile) Ebola response measured by ELISA, VNA, and ELISpot post vaccination (Ad26.ZEBOV followed by MVA-BN-Filo at 56-day interval) at the durability timepoint (last observed titer/concentration obtained ≥4 months post last vaccination) for participants from Africa versus the United States and United Kingdom with boxes representing 25th percentile to 75th percentile. Whiskers extend to the lowest and highest value that is within 1.5 times the interquartile range. The individual participant’s datapoints are plotted over the boxplot as jittered dots. The corresponding table provides an overview of the total participants (N) and the median response and interquartile ranges. Participants were seronegative if the Ad26 NAb titer (IC90) was <LLOQ and seropositive if the Ad26 NAb titer (IC90) was ≥LLOQ. The magnitude of positive Ad26 NAb titers was demarcated into 3 ranges: ≤200 (low), >200 to ≤1000 (moderate), and >1000 (high). African countries included Burkina Faso, Côte d’Ivoire, Kenya, Sierra Leone, Tanzania, and Uganda. ELISA enzyme-linked immunosorbent assay, ELISpot enzyme-linked immunospot, LLOQ lower limit of quantitation, NAb neutralizing antibody, PBMC peripheral blood mononuclear cell, SFU spot forming units, VNA virus neutralization assay.

In participants who were seronegative for Ad26 NAbs at baseline, differences in Ebola vaccine-induced immune responses (post Ad26.ZEBOV vaccination followed by MVA-BN-Filo) were observed based on their geographic region; the median ELISA peak and response durability were lower in Africa (5131 and 397 ELISA units [EU]/mL, respectively) compared to the United States and United Kingdom (10,094 and 1712 EU/mL, respectively; Figs. 2 and 3). Thus, data were split by geographic region to evaluate the role of naturally occurring Ad26 NAbs on the immunogenicity of Ad26-based vaccines.

For Ebola ELISA peak responses, there appeared to be a negligible negative trend between increasing Ad26 NAb titer at baseline and Ebola ELISA peak response within Africa (median responses of 5131, 5289, 4074, and 4534 EU/mL for negative, low, moderate, and high positive Ad26 NAb titers (IC_90_), respectively; Fig. 2). When participants were stratified by Ad26 NAb status at baseline (seropositive or seronegative), median Ebola ELISA peak responses were comparable between Ad26 baseline seronegative and all seropositive participants (low, moderate or high) in Africa (5131 and 4967 EU/mL respectively). For Ebola ELISA, median durability of the response was numerically higher in Ad26 baseline seronegative individuals (397 EU/mL) compared to all those seropositive within Africa (267 EU/mL; Fig. 3). However, there was no negative trend observed with further increasing Ad26 NAb titer and Ebola ELISA durability within Africa (median durability of the responses were 288, 226, and 238 EU/mL for low, moderate, and high positive Ad26 NAb titers [IC_90_], respectively).

The number of peak and durability observations for Ebola VNA and ELISpot was low, especially in the baseline seronegative and high seropositive groups in Africa (*n* ≤ 6), limiting interpretability of the results (Figs. 2 and 3). In the United States and United Kingdom, there was only one participant with a positive Ad26 NAb titer at baseline making this dataset insufficient for further analysis.

### Association between naturally occurring Ad26 NAbs and HIV vaccine-induced responses

**Fig. 4:**
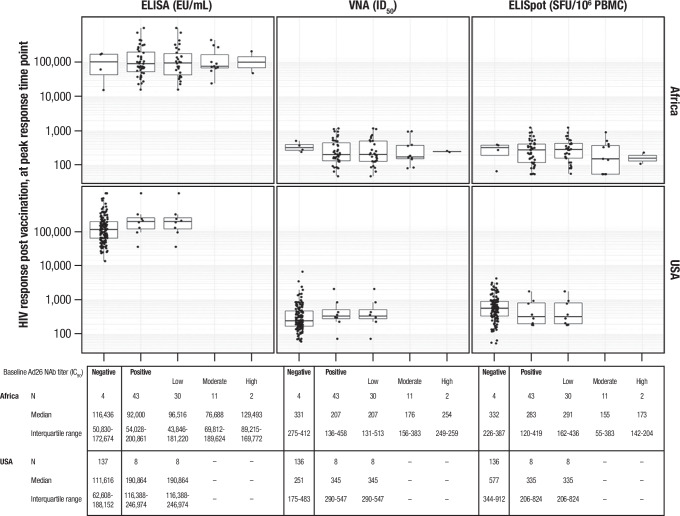
Vaccine-induced peak immune responses post HIV vaccine regimen. Boxplots presenting the median (50th percentile) HIV response measured by ELISA, VNA, and ELISpot post vaccination (4 doses of Ad26.MOS4.HIV with/without protein/adjuvant at Weeks 0, 12, 24, and 48) at the peak timepoint (maximum observed titer/concentration observed <4 months post last vaccination) for participants from Africa versus the United States with boxes representing 25th percentile to 75th percentile. Whiskers extend to the lowest and highest value that is within 1.5 times the interquartile range. The individual participant’s datapoints are plotted over the boxplot as jittered dots. The corresponding table provides an overview of the total participants (N) and the median response and interquartile ranges. Participants were seronegative if the Ad26 NAb titer (IC90) was <LLOQ and seropositive if the Ad26 NAb titer (IC90) was ≥LLOQ. The magnitude of positive Ad26 NAb titers was demarcated into 3 ranges: ≤200 (low), >200 to ≤1000 (moderate), and >1000 (high). African countries included Kenya and Rwanda. ELISA enzyme-linked immunosorbent assay, ELISpot enzyme-linked immunospot, LLOQ lower limit of quantitation, NAb neutralizing antibody, PBMC peripheral blood mononuclear cell, SFU spot forming units, VNA virus neutralization assay.

Analysis of HIV vaccine-induced peak responses within Africa (post four doses of Ad26.Mos4.HIV with/without clade C gp140 protein), was limited to four seronegative participants and two participants with a high Ad26 NAb titer at baseline (Fig. 4). Consistently, median HIV ELISA, VNA, and ELISpot peak responses were higher in baseline Ad26 seronegative participants (116,436 EU/mL, 331 ID_50_, and 332 SFU/10^6^ PBMC, respectively) compared to seropositive participants (92,000 EU/mL, 207 ID_50_, and 283 SFU/10^6^ PBMC, respectively).

**Fig. 5:**
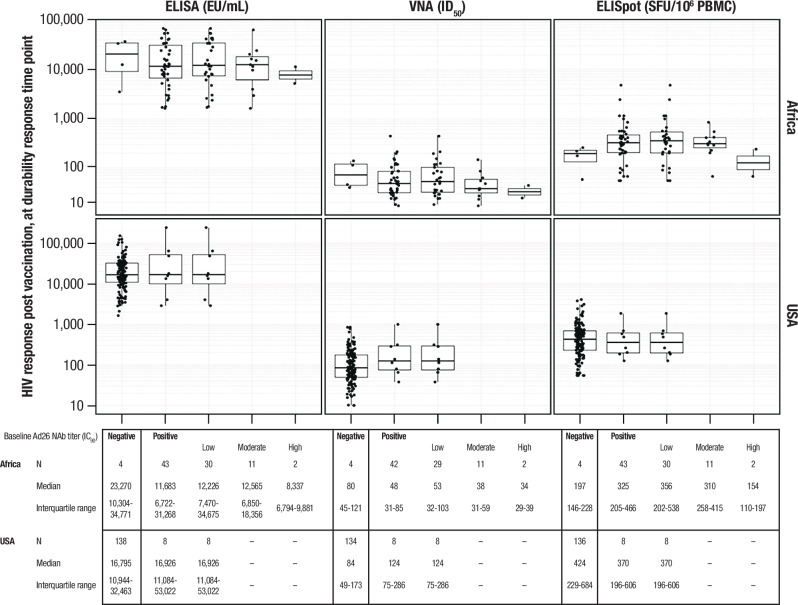
Vaccine-induced durability of the immune responses post HIV vaccine regimen. Boxplots presenting the median (50th percentile) HIV response measured by ELISA, VNA, and ELISpot post vaccination (4 doses of Ad26.MOS4.HIV with/without protein/adjuvant at Weeks 0, 12, 24, and 48) at the durability timepoint (last observed titer/concentration obtained ≥4 months post last vaccination) for participants from Africa versus the United States with boxes representing 25th percentile to 75th percentile. Whiskers extend to the lowest and highest value that is within 1.5 times the interquartile range. The individual participant’s datapoints are plotted over the boxplot as jittered dots. The corresponding table provides an overview of the total participants (N) and the median response and interquartile ranges. Participants were seronegative if the Ad26 NAb titer (IC90) was <LLOQ and seropositive if the Ad26 NAb titer (IC90) was ≥LLOQ. The magnitude of positive Ad26 NAb titers was demarcated into 3 ranges: ≤200 (low), >200 to ≤1000 (moderate), and >1000 (high). African countries included Kenya and Rwanda. ELISA enzyme-linked immunosorbent assay, ELISpot enzyme-linked immunospot, LLOQ lower limit of quantitation, NAb neutralizing antibody, PBMC peripheral blood mononuclear cell, SFU spot forming units, VNA virus neutralization assay.

Durability of the HIV response appeared to show a similar consistent slight negative trend between increasing Ad26 NAb titer and HIV ELISA; VNA durability responses and median HIV ELISA and VNA durability responses were higher in baseline Ad26 seronegative participants (23,270 EU/mL and 80 ID_50_, respectively) compared to seropositive participants (11,683 EU/mL and 80 ID_50_, respectively; Fig. 5). No such consistent association was observed for Ad26 NAb titer and HIV ELISpot responses.

There was no consistent association between Ad26 NAb titer and HIV peak or durability of the response in the United States. While median HIV ELISA and VNA peak responses were lower (111,616 EU/mL and 251 ID_50_ vs 190,864 EU/mL and 345 ID_50_, respectively), ELISpot peak responses were higher (577 vs 335 SFU/10^6^ PBMC, respectively) in baseline Ad26 seronegative compared to seropositive participants (Fig. 4). For durability, median HIV ELISA and ELISpot responses were higher (16,795 EU/mL and 424 SFU/10^6^ PBMC vs 16,926 EU/mL and 370 SFU/10^6^ PBMC, respectively) while durability of the HIV VNA response was lower (84 ID_50_ vs 124 ID_50_, respectively) in baseline Ad26 seronegative compared to seropositive participants (Fig. 5).

### Impact of a previous Ad26-based vaccination

**Fig. 6:**
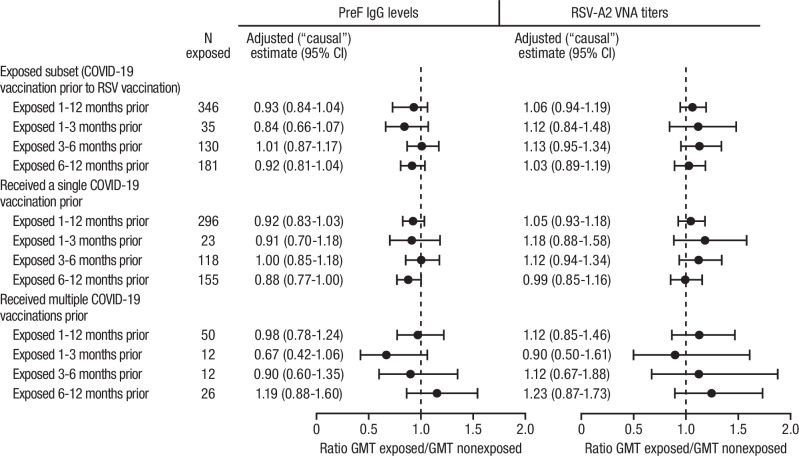
Estimates of the effect of prior COVID-19 vaccination on RSV vaccination. Estimates of the effect with 95% CI of prior COVID-19 vaccination (Ad26.COV2.S) on RSV PreF IgG levels and RSV-A2 VNA titers measured on Day 15 post RSV vaccination (Ad26.RSV.preF/protein). Adjusted (“causal”) estimates are corrected for baseline imbalance in demographic factors and baseline characteristics between the exposed and control groups using inverse probability weighting. The weights were obtained from logistic regression modeling using the SuperLearner ensemble methodology. For analyses with ≥200 exposed participants, predictions were obtained using cross-validation; for analyses with <200 exposed participants, within-sample predictions were used. Four hundred forty-one participants were used for the control group. GMT geometric mean titer, IgG immunoglobulin G, VNA virus neutralization assay.

The impact of previous Ad26-based vaccination on the immunogenicity of a subsequent Ad26-based vaccination, targeting different viruses, was studied post RSV vaccination (Ad26.RSV.preF/protein) in participants previously who received ≥1 COVID-19 vaccinations (Ad26.COV2.S). The ratio of the RSV preF IgG levels at Day 15 post RSV vaccination of participants previously receiving a COVID-19 vaccination (1–12 months prior to RSV) compared to unexposed participants was 0.93 (95% CI 0.84–1.04) (Fig. 6 and Supplementary Fig. S6). Timing of prior exposure appeared to play a negligible role; ratios ranged from 0.84 for prior exposure within 1–3 months to 1.01 for within 3–6 months and 0.92 for within 6–12 months. Several participants received more than one dose of COVID-19 vaccination and showed a minor negative shift of timing (ratios 0.67, 0.90, and 1.19 when the last COVID-19 vaccination dose received was 1–3, 3–6, and 6–12 months prior to RSV, respectively); however, no such trend was observed in participants receiving only one dose of COVID-19 vaccination. Similar results were obtained for Day 15 RSV-A2 VNA titers (Fig. 6 and Supplementary Fig. S7).

The 95% CI of the exposed/unexposed ratios for all analysis (sub)groups included “1”, therefore the study did not detect any statistically significant causal effect of prior exposure to COVID-19 vaccination on Day 15 RSV preF IgG levels and Day 15 RSV-A2 VNA titers post RSV (Fig. 6 and Supplementary Figs. S6 and S7). To take into account naturally acquired RSV responses at baseline, fold-rise responses were also calculated for RSV preF IgG levels and RSV-A2 VNA titers, which showed similar results compared to GMTs (Supplementary Figs. S8 and S9).

**Fig. 7:**
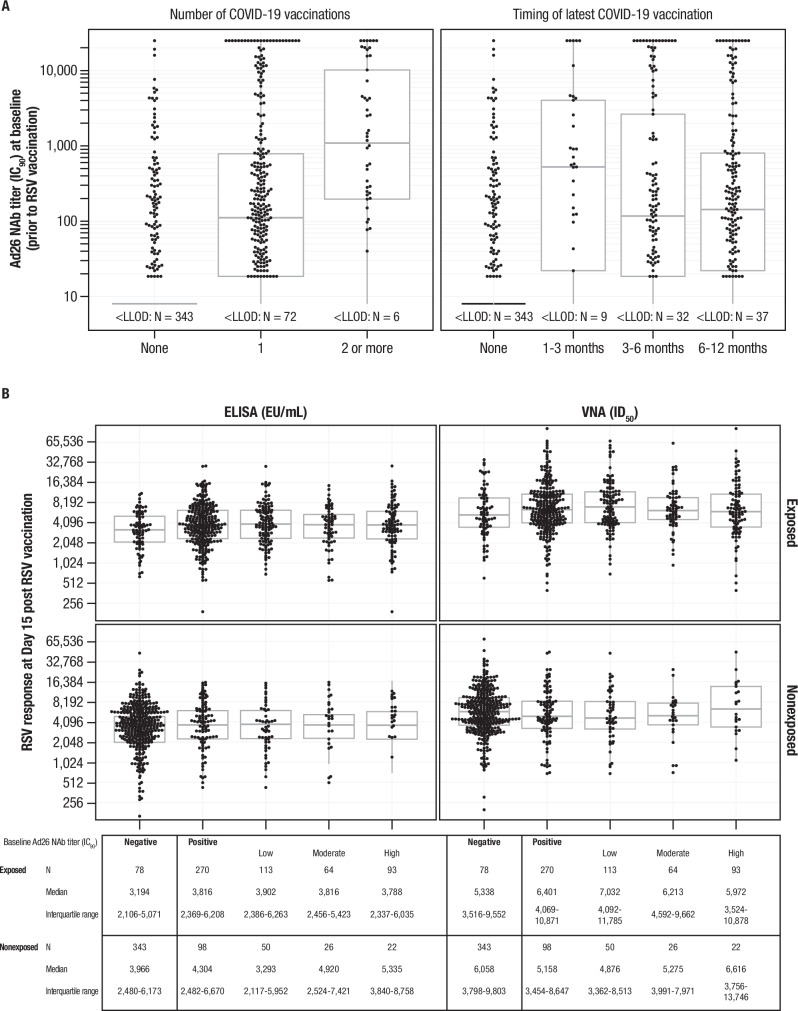
Ad26 NAb titers at baseline prior to RSV vaccination and vaccine-induced immune responses post RSV vaccination. **A** Boxplots showing the naturally occurring Ad26 NAb titers measured at baseline (prior to RSV vaccination) split by the number of previously received Ad26.COV2.S vaccinations and the timing of their last received Ad26.COV2.S vaccination. **B** Boxplots presenting the median (50th percentile) RSV response measured by ELISA and VNA at Day 15 post RSV vaccination (Ad26.RSV.preF/protein) for participants who received an Ad26.COV2.S vaccination 1–12 months prior (exposed) and participants who did not previously receive any adeno-based vaccination (unexposed) with boxes representing 25th percentile to 75th percentile. Whiskers extend to the lowest and highest value that is within 1.5 times the interquartile range. The individual participant’s datapoints are plotted over the boxplot as jittered dots. The corresponding table provides an overview of the total participants (N) and the median response and interquartile ranges. Participants were seronegative if the Ad26 NAb titer (IC90) was <LLOD (16) and seropositive if the Ad26 NAb titer (IC90) was ≥LLOD. The magnitude of positive Ad26 NAb titers was demarcated into 3 ranges: ≤200 (low), >200 to ≤1000 (moderate), and >1000 (high). ULOQ of Ad26 VNA is 24943. ELISA enzyme-linked immunosorbent assay, LLOD lower limit of detection, NAb neutralizing antibody, VNA virus neutralization assay.

Next, the relationship between Ad26 NAb titers at baseline and RSV preF IgG levels and RSV-A2 VNA titers at Day 15 post vaccination was explored. As expected, participants with prior exposure to COVID-19 vaccination had higher Ad26 NAb titers compared to unexposed participants (Fig. 7). Within the exposed participants, those who received multiple COVID-19 vaccinations had higher Ad26 NAb titers compared to those who received only one dose. Participants with prior exposure within 1–3 months tended to have higher Ad26 NAb titers compared with participants with prior exposure within 3–6 and 6–12 months. For both RSV preF IgG levels and RSV-A2 VNA titers, GMT as well as fold-rises, the response at Day 15 post RSV vaccination appeared to be similar between participants who were Ad26 seronegative at baseline and those who were Ad26 seropositive at baseline. There was no observed relationship between elevated Ad26 NAb titer at baseline and the RSV preF IgG levels and RSV-A2 VNA titers, GMT as well as fold-rises, at Day 15 post RSV vaccination (Fig. 7 and Supplementary Fig. S10).

## Discussion

Natural Ad26 NAb seroprevalence was studied by combining pre-vaccination Ad26 NAb data from 21 clinical studies, generating a unique dataset of 3851 study participants from 15 different countries across four continents, with information on participant sex, BMI, and age. This dataset was created before general use of the Ad26-based vaccines for diseases such as COVID-19 and Ebola and therefore represents natural Ad26 NAb seroprevalence. In agreement with earlier published literature, we observed low Ad26 seroprevalence in the United States and Europe and a high seroprevalence in Africa^7–9^. In addition to the dataset presented here, we previously reported a seroprevalence for Ad26 NAbs of 31% in Brazil and 66% in South Africa^28^ and in Japan, 4% of adults aged 20–55 years and 26.3% of adults aged >65 years have Ad26 NAbs^31^. Unpublished data from a Janssen vaccine study (NCT04505722) showed an Ad26 NAb seroprevalence of 39% in Colombia, 78% in Peru, 36% in Argentina, 32% in Chile, and 79% in Mexico (Table S2). This uniquely broad and deep assessment of natural Ad26 seroprevalence across many unstudied geographies confirms that Ad26 seroprevalence varies strongly between different countries.

When assessing the impact of naturally occurring Ad26 NAbs on the immunogenicity of two Ad26-based vaccine regimens, we observed an inconsistent negligible trend for both Ebola and HIV insert-specific immune responses. Interestingly, in this analysis, the difference in vaccine immunogenicity based on geographic region (Africa versus the United States and United Kingdom) was of similar or higher magnitude as the difference observed for Ad26 seropositivity and NAb titers. Factors linked to geographic region that are known to affect vaccine immunogenicity are genetics, nutritional status, the microbiome, while infection with microorganisms and parasites and these could also play a role^57^.

In a clinical study assessing Ad26.COV2.S in Africa, Brazil, and the United States^28^, a negligible correlation was observed between Ad26 NAb titers at baseline and Day 29 S-ELISA responses post vaccination. In Brazil, lower S-ELISA responses were observed in baseline Ad26 seropositive participants, while in South Africa, slightly higher S-ELISA titers were observed in Ad26 seropositive compared to seronegative participants suggesting that geographic or other confounding factors may be involved. Another clinical study assessing a single and 2-dose Ad26.COV2.S regimen in participants in Japan reported no impact of baseline Ad26 NAbs^31^. Negligible negative correlations were observed when impact of Ad26 NAbs was assessed in children and adolescents (aged 1–17 years) from Africa receiving an Ad26-based Ebola vaccine regimen^34,35^. Overall, the presence of naturally occurring Ad26 NAbs at baseline did not have an impact on vaccine-induced insert-specific responses post Ad26-based vaccination. Possibly the level of Ad26 NAbs or their affinity may be too low to neutralize all the Ad26-vectors in the vaccine before they can transduce cells and express the transgene.

Recently, a phase 3 study assessing a multi-dose Ad26-based HIV regimen showed a lower binding antibody response post third vaccination in participants who were Ad26 seropositive at baseline compared to those who were Ad26 seronegative at baseline. Participants included in the immunogenicity analysis were from Argentina, Brazil, Mexico, and Peru^58^. Studies performed in Africa and the United States assessing a similar multidose Ad26-based HIV vaccine did not show any impact of Ad26 NAbs^44,45^. In the phase 3 study, high Ad26 NAb titers (>1000 IC_90_) were observed in a relatively high number of participants (37/177), which could indicate more recent or ongoing Ad26 infections in this population^58^. However, no impact of such high Ad26 NAb titers was detected in studies assessing an Ad26-based Ebola regimen in Africa^1,34–36^. Further research would be required to explore the role of Ad26 NAbs and other confounding factors on the Ad26-based vaccinations in South America.

The potential biological relevance in terms of vaccine efficacy of any observed differences in vaccine-induced, insert-specific immune response based on Ad26 seropositivity remains to be determined. A publication assessing correlates of risk of moderate to severe-critical COVID-19 and correlates of protection of a single-dose Ad26.COV2.S vaccine in the placebo-controlled phase of an international, randomized efficacy trial, determined a hazard ratio of 0.49 (95% CI: 0.29-0.81; *p* = 0.006) per 10-fold increase in spike protein ID_50_ neutralizing titer as measured by psVNA^59^. Thus, the observed slight negative association between Ad26 seropositivity and NAb titers and the vaccine-induced insert-specific immune responses post vaccination for the Ad26-based Ebola and HIV vaccine regimens in this analysis were small relative to the identified associations between immunogenicity and clinical outcomes, albeit for a different antigen, and are unlikely to result in a detectable impact on vaccine effectiveness.

Anti-vector immunity could play a role in homologous multi-dose vaccination regimens and in subsequent vaccination with another vaccine based on the same vector-backbone. We observed that repeated administration of Ad26-based vaccines with the same insert induced immune responses despite the presence of vaccine-induced vector immunity. A 2-dose homologous regimen was explored for an Ad26-based Zika vaccine (29-day interval)^50^, an Ad26-based COVID-19 vaccine (56-day interval)^29–31^, and an Ad26-based RSV vaccine (1-year interval)^49^, and all showed an increase in insert-specific immune response post second vaccination. For a Ad26-based HIV regimen, consisting of four consecutive Ad26-based vaccinations, no consistent correlation was observed between Ad26 NAbs and vaccine insert-specific responses^43–45^. Furthermore, a homologous booster vaccination increased the insert-specific immune response when administered after 6 months in cases of Ad26-based COVID-19 vaccine^53^, or 4 months or 3–5 years for an Ad26-based Ebola vaccine^38,51,52^. Another clinical study assessing both a homologous and heterologous Ad26-based COVID-19 booster found no negative correlation between pre-boost Ad26 NAbs and post boost spike protein NAbs^33^. Overall, irrespective of the presence of Ad26 NAbs, repeated vaccination with a homologous Ad26-based vaccine increased the insert-specific immune response. Since insert-specific immunity was established after the first vaccination (memory response), subsequent boosting seems to be less likely to be impacted by Ad26 NAbs; however, further studies are recommended to understand the exact mechanism.

In addition to repeated vaccination using the same insert, we also report human clinical data following repeated vaccination with Ad26-based vaccines encoding two different inserts. An Ad26-based COVID-19 vaccination received 1–12 months prior to an RSV vaccine had no meaningful impact on the RSV preF protein binding and RSV NAbs measured at Day 15 post RSV vaccination. This is consistent with previously published results in nonhuman primates where several combinations of one or multiple doses of Ad26.RSV.FA2, Ad26.ZEBOV, Ad26.SUDV, and Ad26.HIV.Mos4 were administered at different intervals and did not reveal consistent differences in humoral or cellular responses compared to responses in naïve animals^60^.

This study has several limitations. The univariate analysis presented here could be limited if there are additional confounding factors not already taken into consideration in this analysis that might play a role in immunogenicity. In the case of the HIV vaccination regimen, in most studies there was an additional protein component included in the third and fourth vaccination administration, which might outbalance a possible negative effect of Ad26 NAbs. Combining data from different studies has the advantage of increasing the total sample size, but in the case of the Ebola immunogenicity dataset, the EBOV GP–binding antibody responses post Ad26.ZEBOV vaccination were generated using three versions of the EBOV GP FANG ELISA for which comparability has not been formally demonstrated. Further potential limitations that may affect our observed trends are the heterogeneity across trials in terms of the populations studied, vaccine administered, and assay variability. Because of the heterogeneity in study designs, the need for multiple comparisons, and the post hoc design, statistically significant findings could have occurred by chance alone, justifying the exploratory nature of the analysis, which however, does not allow us to draw statistical conclusions. We addressed heterogeneity in the prevalence dataset by presenting the data by geographical region, age, sex, and BMI. In case of the immunogenicity datasets, only adult data were included and data were presented per “region”. Comparisons were only reported for Ebola, HIV or RSV responses individually. Another limitation of this analysis was that the positive/negative threshold of the Ad26 VNA used to determine the Ad26 NAb titers was dependent on the LLOQ/LLOD. The ranges low, moderate, and high were set based on previous experience^7,8^ and demonstrated some biological relevance but ultimately are arbitrary and may not represent the biological threshold for impact on the ensuing immune responses. We limited our assessment of the impact of anti-vector immunity to NAbs based on past experience showing that cellular and binding responses to adenoviruses are non-specific and ubiquitous, making the assessment of potential interference almost impossible (Johnson & Johnson unpublished data). However, cellular and non-neutralizing humoral responses may still contribute to vector performance and we cannot rule out any impact on anti-vector immunity.

To summarize, in the analysis presented here and in agreement with observations in the literature^1,28–53^, there was no consistent impact observed of Ad26 NAbs (naturally occurring or vaccine induced) on the Ad26-based vaccine-induced insert-specific immune response. Within the limitations of the study, our data suggest that Ad26-based vaccines can be used, irrespective of the high seroprevalence of Ad26 NAbs. The Ad26vector vaccine platform may continue to be useful in future situations, if supported by an appropriate benefit-risk ratio.

## Supplementary information

Supplementary information

## Data Availability

The data sharing policy of Janssen Pharmaceutical Companies of Johnson & Johnson is available at https://www.janssen.com/clinical-trials/transparency. As noted on this site, requests for access to the study data can be submitted through Yale Open Data Access [YODA] Project site at http://yoda.yale.edu.
