# Supplementary material for: The role of naturally occurring and vaccine-induced Ad26 neutralizing antibodies in immunogenicity of Ad26-based vaccines
Source: NPJ Vaccines. 2026 May 21;11:161. doi: 10.1038/s41541-026-01488-8 (PMC13462603; doi:10.1038/s41541-026-01488-8)
Supplement: Supplementary file 1 [file 41541_2026_1488_MOESM1_ESM.pdf]

The role of naturally occurring and vaccine-induced Ad26 neutralizing antibodies in immunogenicity of Ad26-based vaccines

Mariska G.M. van Rosmalen, Joris Menten, Daniel J. Stieh, Nadine Salisch, Janine van Duijn, Arangassery Rosemary Bastian, Chelsea McLean, Viki Bockstal, Kim Stuyckens, Macaya Douoguih, An Vandebosch, Hanneke Schuitemaker, Jenny Hendriks

Supplemental material

Contents

Table S1. Detailed overview of studies included in the prevalence, immunogenicity, and repeat adeno datasets 2

Table S2. Unpublished Ad26 NAb prevalence data from a Janssen vaccine study (NCT04505722)........ 4

Figure S1. Overview of all Ebola (left) and HIV (right) immunogenicity data over time. 5

Figure S2. Standardized mean differences in demographic characteristics between exposed and unexposed cohorts before and after weighting 6

Figure S3. Ad26 NAb titer (IC_{90}) at baseline by sex split per region. 7

Figure S4. Ad26 NAb titer (IC_{90}) at baseline by BMI split per region. 8

Figure S5. Ad26 NAb titer (IC_{90}) at baseline by age split per region. 9

Figure S6. Estimates of the effect of prior COVID-19 vaccination (Ad26.COV.2S) on RSV PreF IgG levels measured on Day 15 post RSV vaccination (Ad26.RSV.preF/protein). 10

Figure S7. Estimates of the effect of prior COVID-19 vaccination (Ad26.COV.2S) on RSV-A2 VNA titers measured on Day 15 post RSV vaccination (Ad26.RSV.preF/protein). 11

Figure S8. Estimates of the effect of prior COVID-19 vaccination (Ad26.COV.2S) on RSV PreF IgG levels fold-rise from baseline to Day 15 post RSV vaccination (Ad26.RSV.preF/protein). 12

Figure S9. Estimates of the effect of prior COVID-19 vaccination (Ad26.COV.2S) on RSV-A2 VNA titers fold-rise from baseline to Day 15 post RSV vaccination (Ad26.RSV.preF/protein). 13

Figure S10. Fold-rise in vaccine-induced immune responses post RSV vaccination. 14

References 15

Table S1. Detailed overview of studies included in the prevalence, immunogenicity, and repeat adeno datasets

Vaccine program	Study no.<br>Refence NCT no.[a]	Vaccine regimen	Phase, blind, allocation	Country	Study population, age in years	No. of participants in datasets		
						Prevalence	Ebola and HIV immunogenicity	Repeat adeno
Ebola	VAC52150EBL1001<br>NCT02313077[1,2]	Ad26.ZEBOV, MVA-BN-Filo	Phase 1, part 1<br>Double-blind (observer-blind)<br>Randomized	UK	Healthy, 18–50	51	14	-
	VAC52150EBL1002<br>NCT02325050[3]	Ad26.ZEBOV, MVA-BN-Filo	Phase 1<br>Double-blind (observer-blind)<br>Randomized	USA	Healthy, 18–50	63	-	-
	VAC52150EBL1003<br>NCT02376426[4]	Ad26.ZEBOV, MVA-BN-Filo	Phase 1<br>Double-blind (observer-blind)<br>Randomized	Kenya	Healthy, 18–50	36	15	-
	VAC52150EBL1004<br>NCT02376400[5]	Ad26.ZEBOV, MVA-BN-Filo	Phase 1<br>Double-blind (observer-blind)<br>Randomized	Tanzania, Uganda	Healthy, 18–50	35	15	-
	VAC52150EBL2002<br>NCT02564523[6,7]	Ad26.ZEBOV, MVA-BN-Filo	Phase 2<br>Double-blind (observer-blind)<br>Randomized	Burkina Faso, Uganda, Côte d'Ivoire, Kenya	Healthy, 4–70 and HIV+ adults, 18–50	501	99	-
	VAC52150EBL2003<br>NCT02598388	Ad26.ZEBOV, MVA-BN-Filo	Phase 2<br>Double-blind (observer-blind)<br>Randomized	USA, Kenya, Mozambique, Nigeria, Tanzania, Uganda	Healthy, 18–70	176	-	-
	VAC52150EBL3001<br>NCT02509494[8,9]	Ad26.ZEBOV, MVA-BN-Filo	Phase 3<br>Stage 1: Open-label, uncontrolled<br>Stage 2: Double-blind, randomized	Sierra Leone	Stage 1: Healthy, ≥18<br>Stage 2: Healthy, ≥1	880	241	-
	VAC69120FLV1001<br>NCT02860650[10]	Ad26.Filo, Ad26.ZEBOV	Phase 1<br>Double-blind (observer-blind for subset in Group 3 receiving booster)<br>Randomized	USA	Healthy, 18–50	65	11	-
HIV	HIV-V-A004<br>NCT02315703 [11]	Ad26.Mos.HIV	Phase 1/2a<br>Double-blind<br>Randomized	USA, Thailand, Rwanda, Uganda, South Africa	Healthy, 18–50	390	-	-
	VAC89220HPX1002<br>NCT02685020[12]	Ad26.Mos.HIV w/o protein	Phase 1<br>Double-blind<br>Randomized	USA	Healthy, 18–50	36	-	-
	VAC89220HPX2003<br>NCT02935686[13]	Ad26.Mos4.HIV w/o protein	Phase 1/2a<br>Double-blind<br>Randomized	USA, Kenya, Rwanda	Healthy, 18–50	152	101	-
	VAC89220HPX2004<br>NCT02788045[14]	Ad26.Mos.HIV<br>Ad26.Mos4.HIV w/o protein	Phase 1/2a<br>Double-blind<br>Randomized	USA, Rwanda	Healthy, 18–50	198	93	-
RSV	VAC18192RSV1001<br>NCT02440035	Ad35.RSV.FA2, Ad26.RSV.FA2	Phase 1<br>Double-blind (observer-blind)<br>Randomized	USA	Healthy, 18–50	44	-	-

Vaccine program	Study no. Refence NCT no.[a]	Vaccine regimen	Phase, blind, allocation	Country	Study population, age in years	No. of participants in datasets		
						Prevalence	Ebola and HIV immunogenicity	Repeat adeno
	VAC18192RSV1003 NCT02926430	Ad35.RSV.FA2, Ad26.RSV.FA2	Phase 1 Double-blind (observer-blind) Randomized	USA	Healthy, ≥60	31	-	-
	VAC18193RSV1003 NCT02926430[15]	Ad26.RSV.preF	Phase 1 Double-blind Randomized	USA	Healthy, ≥60	72	-	-
	VAC18193RSV1004 NCT03502707[16]	Ad26.RSV.preF Ad26.RSV.preF /protein	Phase 1/2a Double-blind (observer-blind) Randomized	USA	Healthy, ≥60	626	-	-
	VAC18193RSV1005 NCT03795441	Ad26.RSV.preF	Phase 1 Open label	Belgium	Healthy, ≥18	24	-	-
	VAC18193RSV2001 NCT03982199[17]	Ad26.RSV.preF /protein	Phase 2 Triple-blind (participant, care provider, investigator) Randomized	USA	Healthy, ≥65	194	-	-
	VAC18193RSV2002 NCT03334695[18]	Ad26.RSV.preF	Phase 2a Double-blind (observer-blind) Randomized	UK	Healthy, 18–50	61	-	-
	VAC18193RSV2003 NCT03339713[19]	Ad26.RSV.preF	Phase 2a Double-blind (observer-blind) Randomized	USA	Healthy, ≥60	178	-	-
	VAC18194RSV2001 NCT03303625[20]	Ad26.RSV.preF	Phase 1/2 Double-blind (observer-blind)	Finland, UK, USA	Healthy, 18–50 and RSV–seropositive, 12–24 months of age	38	-	-
	VAC18193RSV3001 NCT04908683	Ad26.RSV.preF /protein	Phase 3 Double-blind (placebo-controlled) Randomized	USA, Australia, Brazil, Canada, Chile, China, Estonia, Finland, New Zealand, Poland, South Africa, Taiwan, Thailand, UK	Healthy, ≥60	-	-	348 exposed and 441 unexposed

HIV, human immunodeficiency virus; UK, United Kingdom; USA, United States of America; w/o, with or without

[a] clinicaltrials.gov

Table S2. Unpublished Ad26 NAb prevalence data from a Janssen vaccine study (NCT04505722)

Country	Ad26 NAb prevalence (participants aged 40–70 years)
Argentina	36% (24/66)
Chile	32% (7/22)
Colombia	39% (57/146)
Mexica	79% (11/14)
Peru	78% (61/78)

Figure S1. Overview of all Ebola (left) and HIV (right) immunogenicity data over time.

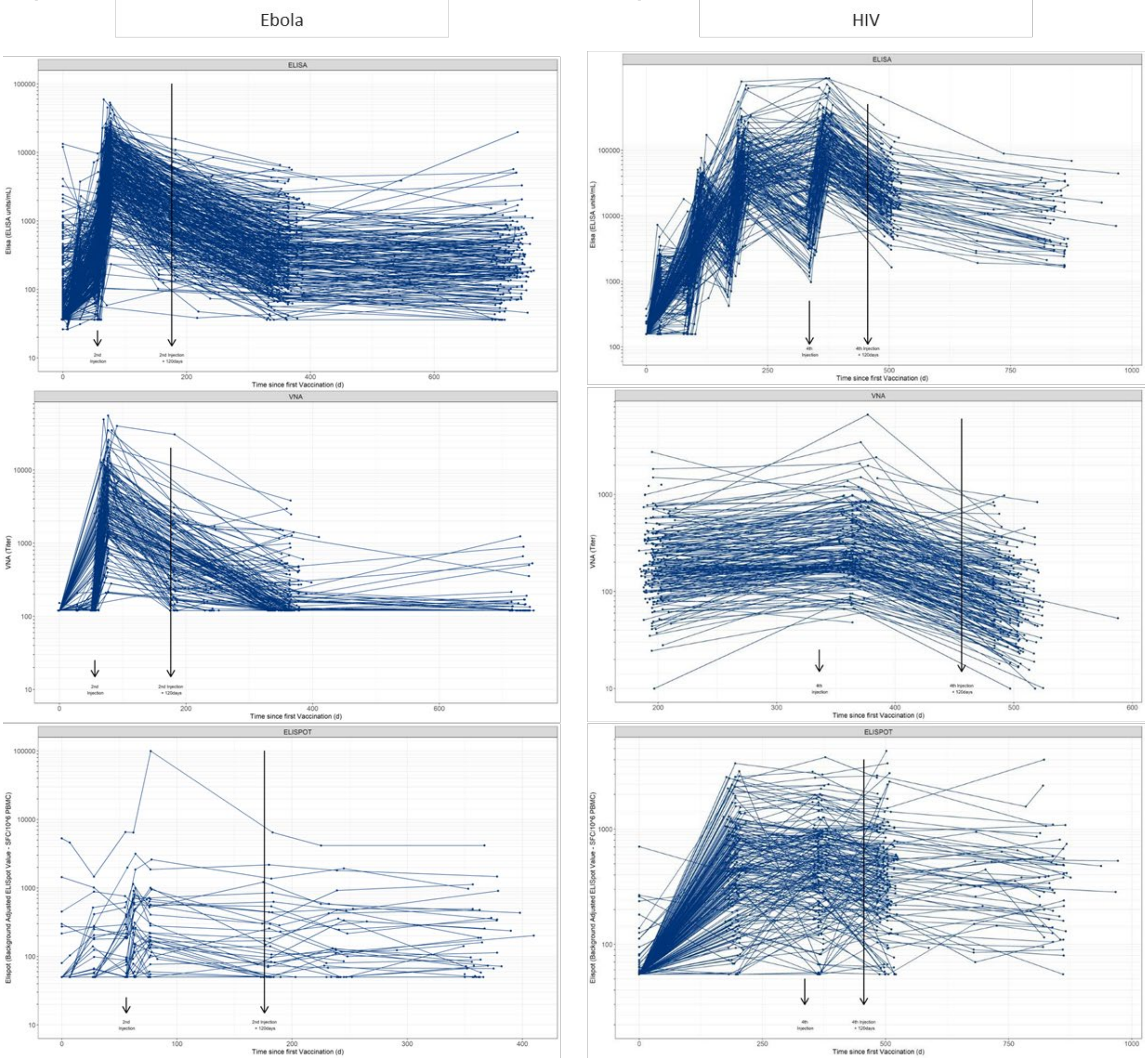

Line plots of all available data points per participant. Participants receiving the Ebola regimen (Ad26.ZEBOV followed by MVA-BN-Filo at a 56-day interval) on the left and the HIV regimen (4 doses of Ad26.MOS4.HIV with/without protein/adjuvant at Weeks 0, 12, 24, and 48) with ELISA, VNA, and ELISpot data from top to bottom. Arrows indicate the target timepoint of last vaccination according to the vaccine regimen (Ebola; 2nd injection is given at Day 57 according to the trial protocol/HIV; 4th injection is given at Day 337 according to the trial protocol) and +120 days representing the 4-month cutoff used to distinguish between peak and durability timepoints.

ELISA, enzyme-linked immunosorbent assay; ELISpot, enzyme-linked immunospot; PBMC, peripheral blood mononuclear cell; SFC, spot forming cells; VNA, virus neutralization assay.

Figure S2. Standardized mean differences in demographic characteristics between exposed and unexposed cohorts before and after weighting

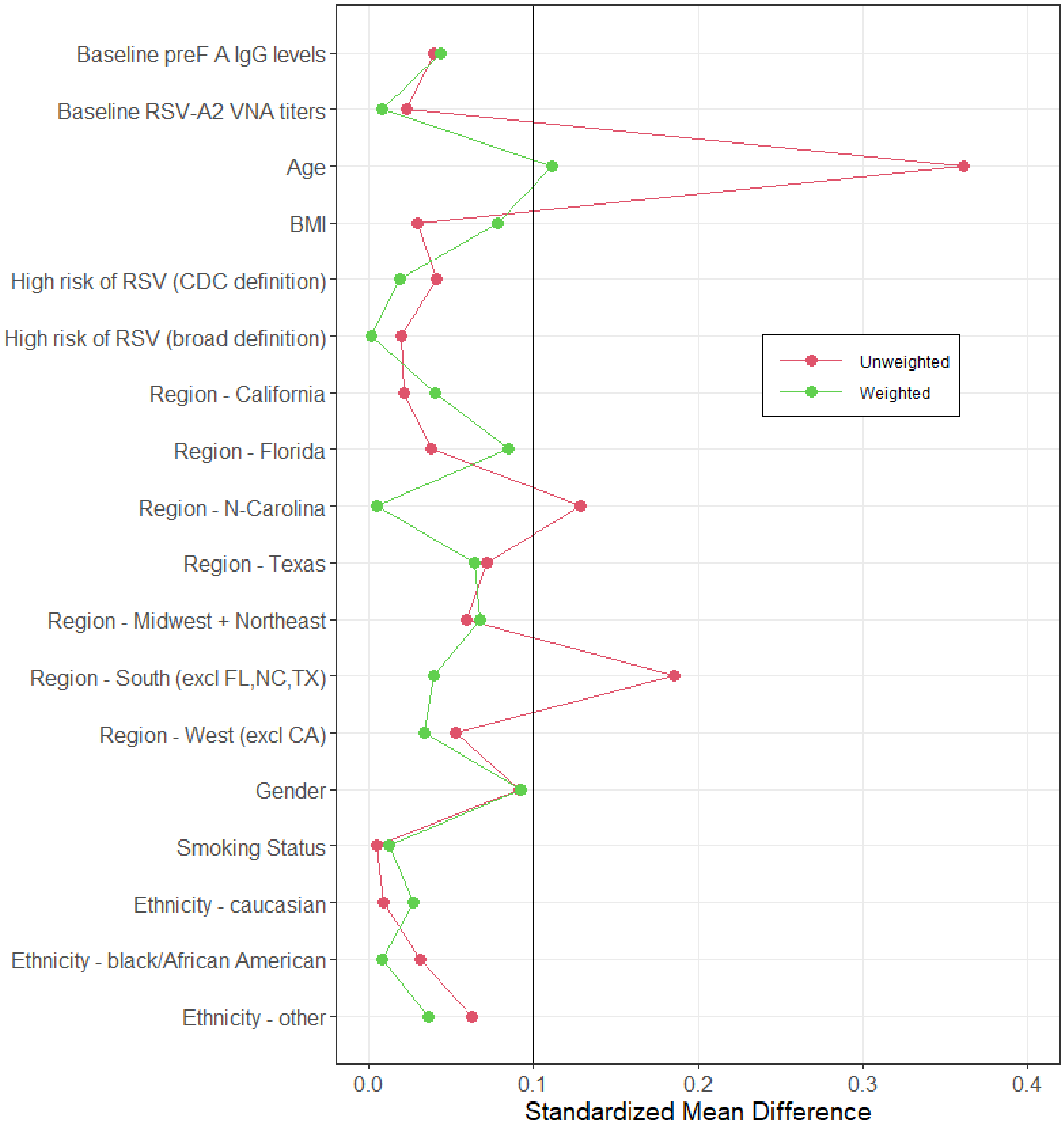

BMI, body mass index; CDC, US Centers for Disease Control and Prevention; RSV, respiratory syncytial virus; N, North

Narrow definition: Chronic obstructive pulmonary disease, asthma, congestive heart failure, other chronic heart and lung disease.

Broad definition: any of the above, or chronic kidney disease and diabetes.

Figure S3. Ad26 NAb titer (IC_{90}) at baseline by sex split per region.

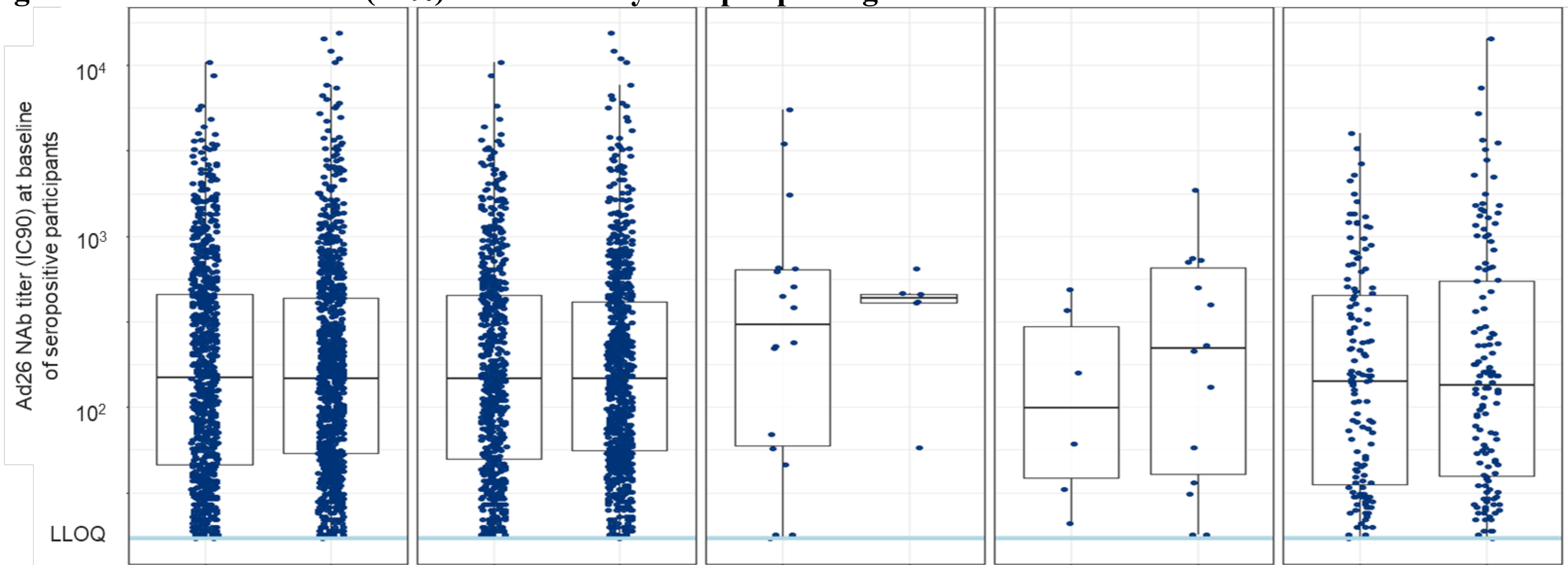

Continent	All		Africa		Asia		EU		N.America	
Sex	Female	Male	Female	Male	Female	Male	Female	Male	Female	Male
N	1913	1938	797	1075	29	29	84	89	1003	745
% pos	42	50	80	76	62	21	7	16	13	18
(95% CI)	(39;44)	(48;52)	(77;83)	(73;79)	(42;79)	(9;40)	(3;16)	(9;25)	(11;15)	(15;21)
GMT pos	164	164	167	164	244	332	103	172	144	160
(95% CI)	(148;180)	(151;179)	(150;186)	(150;180)	(103;579)	(139;792)	(28;375)	(71;414)	(112;184)	(122;210)

Boxplots showing the median (50th percentile) Ad26 NAb titers of Ad26 seropositive participants with boxes representing 25th percentile to 75th percentile. Whiskers extend to the lowest and highest value that is within 1.5 times the interquartile range. Individual participant datapoints are plotted over the boxplot as jittered and colored dots. The table provides an overview of the total participants (N) and the % Ad26 seropositive (% pos) participants and their respective GMT (GMT pos) by sex split per region. The % seropositive and GMTs are color coded with the lowest value being blue and the highest value being yellow. Participants were Ad26 seropositive if Ad26 NAb titer (IC_{90}) ≥LLOQ.

African countries included Burkina Faso, Côte d'Ivoire, Kenya, Mozambique, Nigeria, Rwanda, Sierra Leone, Tanzania, Uganda, and South Africa. Asian countries included Thailand. EU countries included Belgium, Finland, and the United Kingdom. North America refers exclusively to the United States. GMT, geometric mean titer; LLOQ, lower limit of quantitation; NAb, neutralizing antibody.

Figure S4. Ad26 NAb titer (IC_{90}) at baseline by BMI split per region.

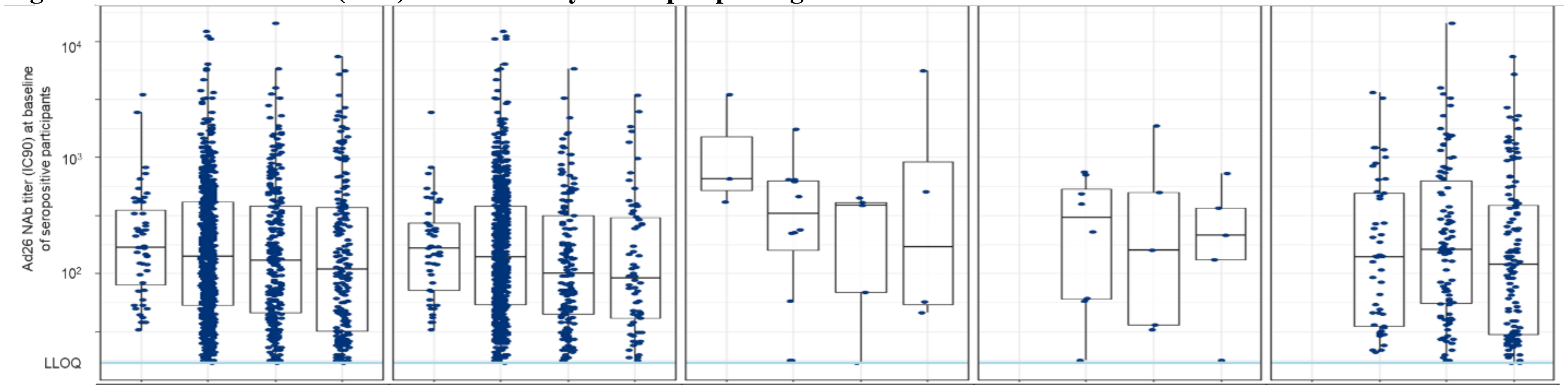

Continent	All				Africa				Asia				EU				N.America			
BMI	Under-weight	Normal weight	Pre-obese	Obese	Under-weight	Normal weight	Pre-obese	Obese	Under-weight	Normal weight	Pre-obese	Obese	Under-weight	Normal weight	Pre-obese	Obese	Under-weight	Normal weight	Pre-obese	Obese
N	68	1438	837	677	57	762	182	67	3	38	11	6	1	79	41	26	7	559	603	578
% pos	79	52	32	28	90	89	89	93	100	32	46	67	0	10	12	19	0	9	16	21
(95% CI)	(68;88)	(49;54)	(29;35)	(25;32)	(78;96)	(86;91)	(83;93)	(83;97)	(31;100)	(18;49)	(18;75)	(24;94)	(0;95)	(5;20)	(5;27)	(7;40)	(0;43.9)	(7;12)	(13;19)	(18;25)
GMT pos	171	150	143	130	154	149	121	117	984	230	153	293	-	187	178	168	-	143	187	132
(95% CI)	(130;226)	(137;165)	(121;169)	(105;161)	(119;200)	(136;164)	(99;147)	(83;165)	(126;7712)	(92;571)	(29;809)	(13;6549)	-	(61;577)	(24;1301)	(34;843)	-	(94;218)	(137;254)	(100;174)

Boxplots showing the median (50th percentile) Ad26 NAb titers of Ad26 seropositive participants with boxes representing 25th percentile to 75th percentile. Whiskers extend to the lowest and highest value that is within 1.5 times the interquartile range. Individual participant datapoints are plotted over the boxplot as jittered and colored dots. The table provides an overview of the total participants (N) and the % Ad26 seropositive (% pos) participants and their respective GMT (GMT pos) by BMI split per region. The % seropositive and GMTs are color coded with the lowest value being blue and the highest value being yellow. The BMI categories studied were defined as follows: underweight (<18 kg/m^2), normal weight (18 to <25 kg/m^2), pre-obese (25 to <30 kg/m^2), and obese (>30 kg/m^2).

African countries included Burkina Faso, Côte d'Ivoire, Kenya, Mozambique, Nigeria, Rwanda, Sierra Leone, Tanzania, Uganda, and South Africa. Asian countries included Thailand. EU countries included Belgium, Finland, and the United Kingdom. North America refers exclusively to the United States. BMI, body mass index; GMT, geometric mean titer; LLOQ, lower limit of quantitation; NAb, neutralizing antibody.

Figure S5. Ad26 NAb titer (IC_{90}) at baseline by age split per region.

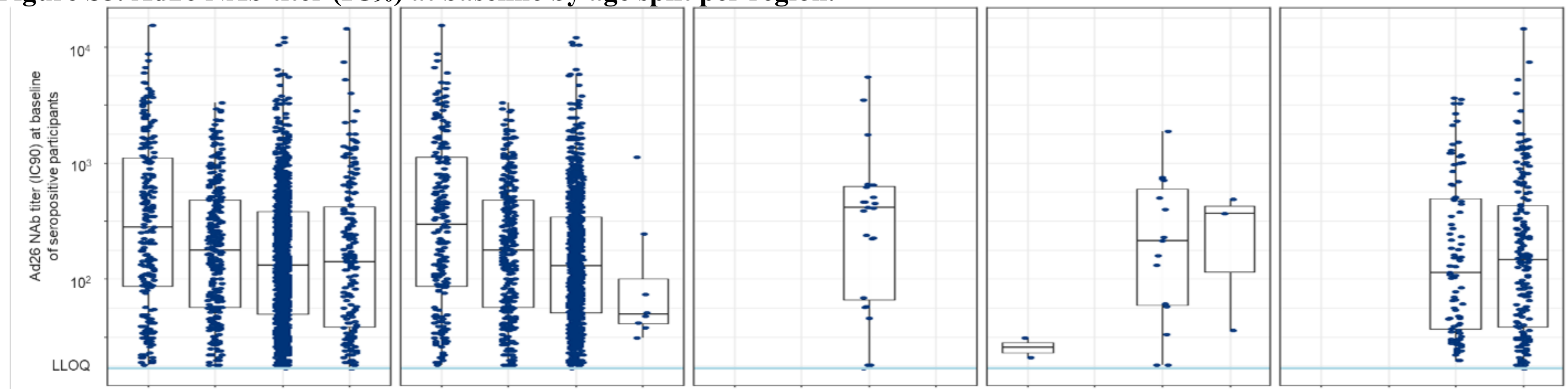

Continent	All			
Age	Children	Adolescents	Adults	Elderly
N	466	365	1933	1087
% pos (95% CI)	47 (42;52)	80 (76;84)	55 (53;58)	17 (15;20)
GMT pos (95% CI)	285 (229;355)	177 (152;206)	146 (135;157)	149 (121;185)

Continent	Africa			
Age	Children	Adolescents	Adults	Elderly
N	439	365	1059	9
% pos (95% CI)	49 (45;54)	80 (76;84)	89 (87;91)	89 (51;99)
GMT pos (95% CI)	292 (234;363)	177 (152;206)	143 (132;155)	84 (31;228)

Continent	Asia			
Age	Children	Adolescents	Adults	Elderly
N			58	
% pos (95% CI)			41 (29;55)	
GMT pos (95% CI)			263 (136;508)	

Continent	EU			
Age	Children	Adolescents	Adults	Elderly
N	26		139	8
% pos (95% CI)	8 (1;27)		11 (6;18)	38 (10;74)
GMT pos (95% CI)	26 (11-59)		178 (80;393)	186 (13;2586)

Continent	N.America			
Age	Children	Adolescents	Adults	Elderly
N	1		677	1070
% pos (95% CI)	0 (0;95)		13 (11;16)	16 (14;19)
GMT pos (95% CI)	-		149 (108;207)	153 (122;191)

Boxplots showing the median (50th percentile) Ad26 NAb titers of Ad26 seropositive participants with boxes representing 25th percentile to 75th percentile. Whiskers extend to the lowest and highest value that is within 1.5 times the interquartile range. Individual participant datapoints are plotted over the boxplot as jittered and colored dots. The table provides an overview of the total participants (N) and the % Ad26 seropositive (% pos) participants and their respective GMT (GMT pos) by age split per region. The % seropositive and GMTs are color coded with the lowest value being blue and the highest value being yellow. Age was divided into the following: categories: children (≥1 to <10 years of age), adolescents (≥10 to <17 years of age), adults (≥17 to <60 years of age, and elderly (≥60 years of age).

African countries included Burkina Faso, Côte d'Ivoire, Kenya, Mozambique, Nigeria, Rwanda, Sierra Leone, Tanzania, Uganda, and South Africa. Asian countries included Thailand. EU countries included Belgium, Finland, and the United Kingdom. North America refers exclusively to the United States. GMT, geometric mean titer; LLOQ, lower limit of quantitation; NAb, neutralizing antibody.

Figure S6. Estimates of the effect of prior COVID-19 vaccination (Ad26.COV.2S) on RSV PreF IgG levels measured on Day 15 post RSV vaccination (Ad26.RSV.preF/protein).

	N exposed	Unadjusted (“naïve”) estimate (96% CI)	Adjusted (“causal”) estimate (96% CI)
Exposed subset (COVID-19 vaccination prior to RSV vaccination)			
exposed 1 – 12 months prior	346	0.96 (0.86, 1.07)	0.93 (0.84, 1.04)
exposed 1 – 3 months prior	35	0.84 (0.64, 1.09)	0.84 (0.66, 1.07)
exposed 3 – 6 months prior	130	1.06 (0.91, 1.24)	1.01 (0.87, 1.17)
exposed 6 – 12 months prior	181	0.92 (0.81, 1.05)	0.92 (0.81, 1.04)
Received a singe COVID-19 vaccination prior			
exposed 1 – 12 months prior	296	0.96 (0.86, 1.07)	0.92 (0.83, 1.03)
exposed 1 – 3 months prior	23	0.93 (0.68, 1.28)	0.91 (0.70, 1.18)
exposed 3 – 6 months prior	118	1.08 (0.92, 1.26)	1.00 (0.85, 1.18)
exposed 6 – 12 months prior	155	0.89 (0.77, 1.02)	0.88 (0.77, 1.00)
Received multiple COVID-19 vaccinations prior			
exposed 1 – 12 months prior	50	0.97 (0.77, 1.23)	0.98 (0.78, 1.24)
exposed 1 – 3 months prior	12	0.68 (0.43, 1.07)	0.67 (0.42, 1.06)
exposed 3 – 6 months prior	12	0.91 (0.58, 1.44)	0.90 (0.60, 1.35)
exposed 6 – 12 months prior	26	1.19 (0.86, 1.64)	1.19 (0.88, 1.60)

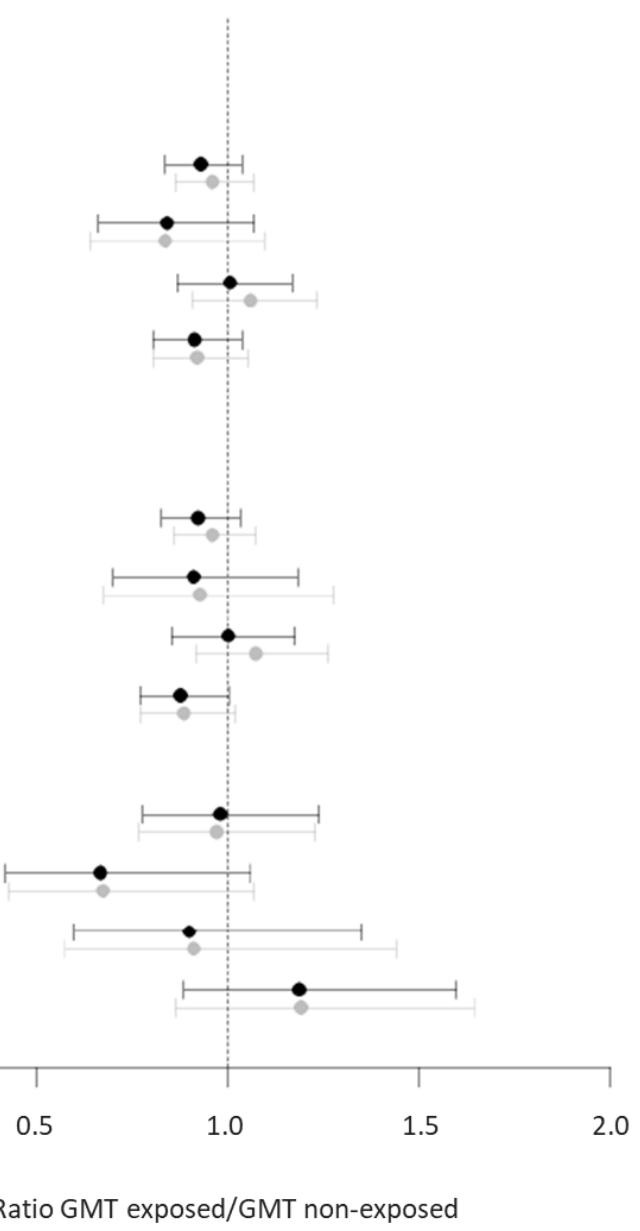

Unadjusted (“naïve”) estimates (grey) are based on a simple comparison of the observed responses in exposed and unexposed groups and are only reported for descriptive purposes. This estimate is expected to be biased as it does not take potential confounders into account. Adjusted (“causal”) estimates (black) are corrected for baseline imbalance in demographic factors and baseline characteristics between the exposed and control groups using inverse probability weighting. The weights were obtained from logistic regression modeling using the SuperLearner ensemble methodology. For analyses with ≥200 exposed participants, predictions were obtained using cross-validation; for analyses with <200 exposed participants within-sample predictions were used. The control group included 441 participants.

GMT, geometric mean titer; IgG, immunoglobulin G.

Figure S7. Estimates of the effect of prior COVID-19 vaccination (Ad26.COV.2S) on RSV-A2 VNA titers measured on Day 15 post RSV vaccination (Ad26.RSV.preF/protein).

	N exposed	Unadjusted ("naïve") estimate (96% CI)	Adjusted ("causal") estimate (96% CI)
Exposed subset (COVID-19 vaccination prior to RSV vaccination)			
exposed 1 – 12 months prior	346	1.10 (0.98, 1.24)	1.06 (0.94, 1.19)
exposed 1 – 3 months prior	35	1.12 (0.85, 1.47)	1.12 (0.84, 1.48)
exposed 3 – 6 months prior	130	1.19 (1.01, 1.39)	1.13 (0.95, 1.34)
exposed 6 – 12 months prior	181	1.04 (0.90, 1.19)	1.03 (0.89, 1.19)
Received a singe COVID-19 vaccination prior			
exposed 1 – 12 months prior	296	1.09 (0.97, 1.24)	1.05 (0.93, 1.18)
exposed 1 – 3 months prior	23	1.24 (0.90, 1.72)	1.18 (0.88, 1.58)
exposed 3 – 6 months prior	118	1.19 (1.01, 1.41)	1.12 (0.94, 1.34)
exposed 6 – 12 months prior	155	1.01 (0.87, 1.17)	0.99 (0.85, 1.16)
Received multiple COVID-19 vaccinations prior			
exposed 1 – 12 months prior	50	1.12 (0.88, 1.42)	1.12 (0.85, 1.46)
exposed 1 – 3 months prior	12	0.90 (0.57, 1.44)	0.90 (0.50, 1.61)
exposed 3 – 6 months prior	12	1.13 (0.71, 1.79)	1.12 (0.67, 1.88)
exposed 6 – 12 months prior	26	1.23 (0.89, 1.70)	1.23 (0.87, 1.73)

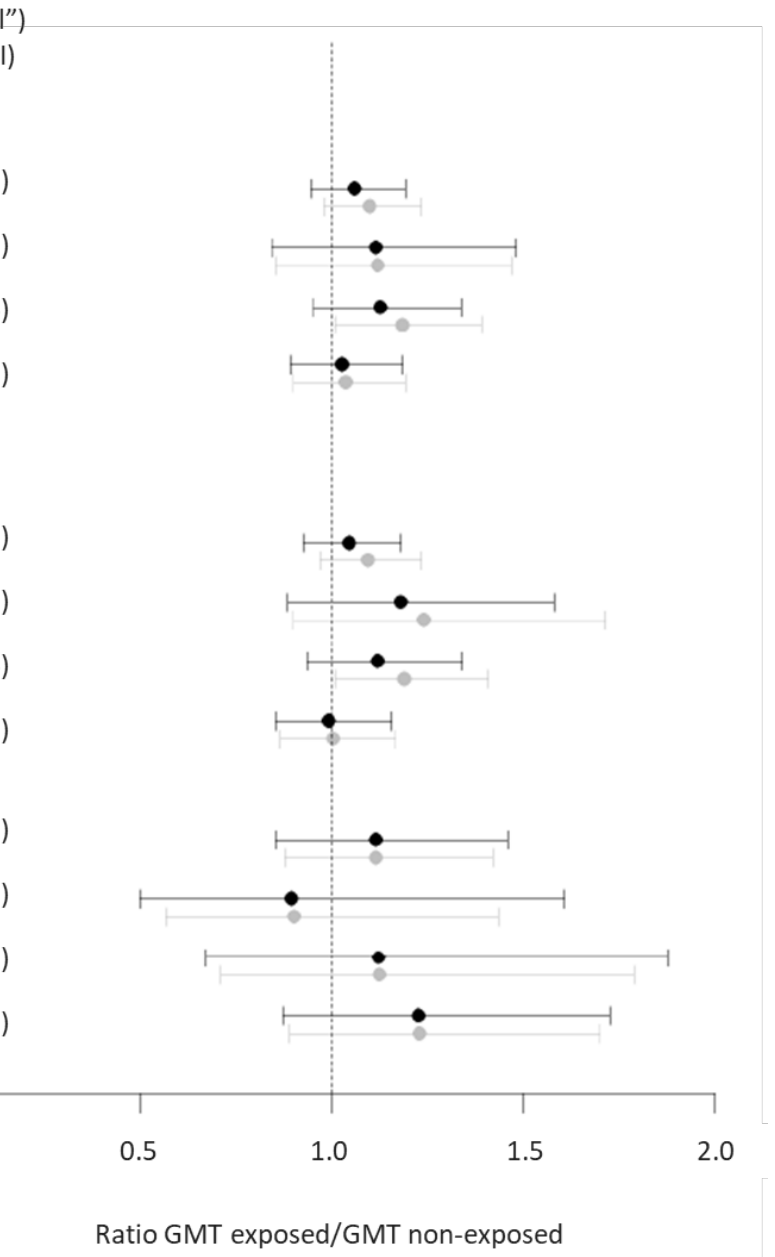

Unadjusted ("naive") estimates (grey) are based on a simple comparison of the observed responses in exposed and unexposed groups and are only reported for descriptive purposes. This estimate is expected to be biased as it does not take potential confounders into account. Adjusted ("causal") estimates (black) are corrected for baseline imbalance in demographic factors and baseline characteristics between the exposed and control groups using inverse probability weighting. The weights were obtained from logistic regression modeling using the SuperLearner ensemble methodology. For analyses with ≥200 exposed participants, predictions were obtained using cross-validation; for analyses with <200 exposed participants within-sample predictions were used. The control group included 441 participants.

GMT, geometric mean titer; VNA, virus neutralization assay.

Figure S8. Estimates of the effect of prior COVID-19 vaccination (Ad26.COV.2S) on RSV PreF IgG levels fold-rise from baseline to Day 15 post RSV vaccination (Ad26.RSV.preF/protein).

	N exposed	Unadjusted (“naïve”) estimate (96% CI)	Adjusted (“causal”) estimate (96% CI)
Exposed subset (COVID-19 vaccination prior to RSV vaccination)			
exposed 1 – 12 months prior	346	0.93 (0.81, 1.07)	0.90 (0.78, 1.04)
exposed 1 – 3 months prior	35	0.92 (0.65, 1.29)	0.91 (0.68, 1.20)
exposed 3 – 6 months prior	130	0.96 (0.78, 1.18)	0.93 (0.75, 1.16)
exposed 6 – 12 months prior	181	0.92 (0.77, 1.09)	0.91 (0.77, 1.08)
Received a singe COVID-19 vaccination prior			
exposed 1 – 12 months prior	296	0.94 (0.81, 1.09)	0.90 (0.78, 1.05)
exposed 1 – 3 months prior	23	1.06 (0.70, 1.60)	0.94 (0.67, 1.31)
exposed 3 – 6 months prior	118	1.00 (0.81, 1.24)	0.96 (0.77, 1.20)
exposed 6 – 12 months prior	155	0.88 (0.73, 1.06)	0.87 (0.73, 1.05)
Received multiple COVID-19 vaccinations prior			
exposed 1 – 12 months prior	50	0.88 (0.65, 1.19)	0.94 (0.69, 1.29)
exposed 1 – 3 months prior	12	0.67 (0.37, 1.21)	0.65 (0.41, 1.01)
exposed 3 – 6 months prior	12	0.62 (0.34, 1.13)	0.70 (0.33, 1.49)
exposed 6 – 12 months prior	26	1.18 (0.78, 1.79)	1.21 (0.84, 1.73)

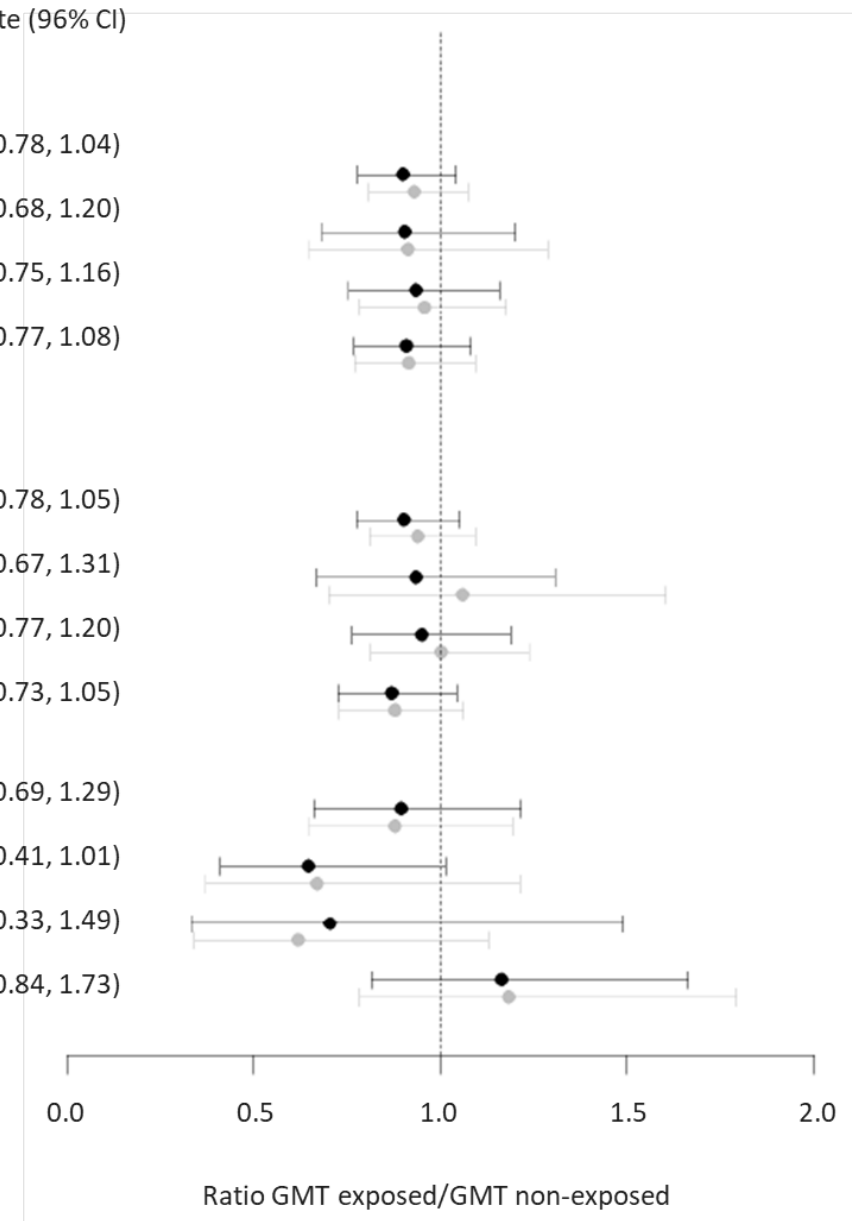

Unadjusted (“naive”) estimates (grey) are based on a simple comparison of the observed responses in exposed and unexposed groups and are only reported for descriptive purposes. This estimate is expected to be biased as it does not potential confounders take into account. Adjusted (“causal”) estimates (black) are corrected for baseline imbalance in demographic factors and baseline characteristics between the exposed and control groups using inverse probability weighting. The weights were obtained from logistic regression modeling using the SuperLearner ensemble methodology. For analyses with ≥200 exposed participants, predictions were obtained using cross-validation; for analyses with <200 exposed participants within-sample predictions were used. The control group included 441 participants.

GMT, geometric mean titer; IgG, immunoglobulin G.

Figure S9. Estimates of the effect of prior COVID-19 vaccination (Ad26.COV.2S) on RSV-A2 VNA titers fold-rise from baseline to Day 15 post RSV vaccination (Ad26.RSV.preF/protein).

	N exposed	Unadjusted (“naïve”) estimate (96% CI)	Adjusted (“causal”) estimate (96% CI)
Exposed subset (COVID-19 vaccination prior to RSV vaccination)			
exposed 1 – 12 months prior	346	1.08 (0.93, 1.26)	1.05 (0.90, 1.23)
exposed 1 – 3 months prior	35	1.14 (0.80, 1.61)	1.13 (0.82, 1.55)
exposed 3 – 6 months prior	130	1.10 (0.89, 1.35)	1.08 (0.86, 1.37)
exposed 6 – 12 months prior	181	1.06 (0.88, 1.27)	1.05 (0.87, 1.27)
Received a singe COVID-19 vaccination prior			
exposed 1 – 12 months prior	296	1.08 (0.92, 1.26)	1.05 (0.89, 1.23)
exposed 1 – 3 months prior	23	1.24 (0.81, 1.89)	1.12 (0.76, 1.64)
exposed 3 – 6 months prior	118	1.14 (0.92, 1.42)	1.11 (0.87, 1.41)
exposed 6 – 12 months prior	155	1.01 (0.83, 1.23)	1.00 (0.82, 1.22)
Received multiple COVID-19 vaccinations prior			
exposed 1 – 12 months prior	50	1.06 (0.78, 1.46)	1.13 (0.80, 1.61)
exposed 1 – 3 months prior	12	0.95 (0.52, 1.73)	0.91 (0.53, 1.56)
exposed 3 – 6 months prior	12	0.75 (0.41, 1.38)	0.86 (0.38, 1.96)
exposed 6 – 12 months prior	26	1.33 (0.87, 2.03)	1.35 (0.87, 2.09)

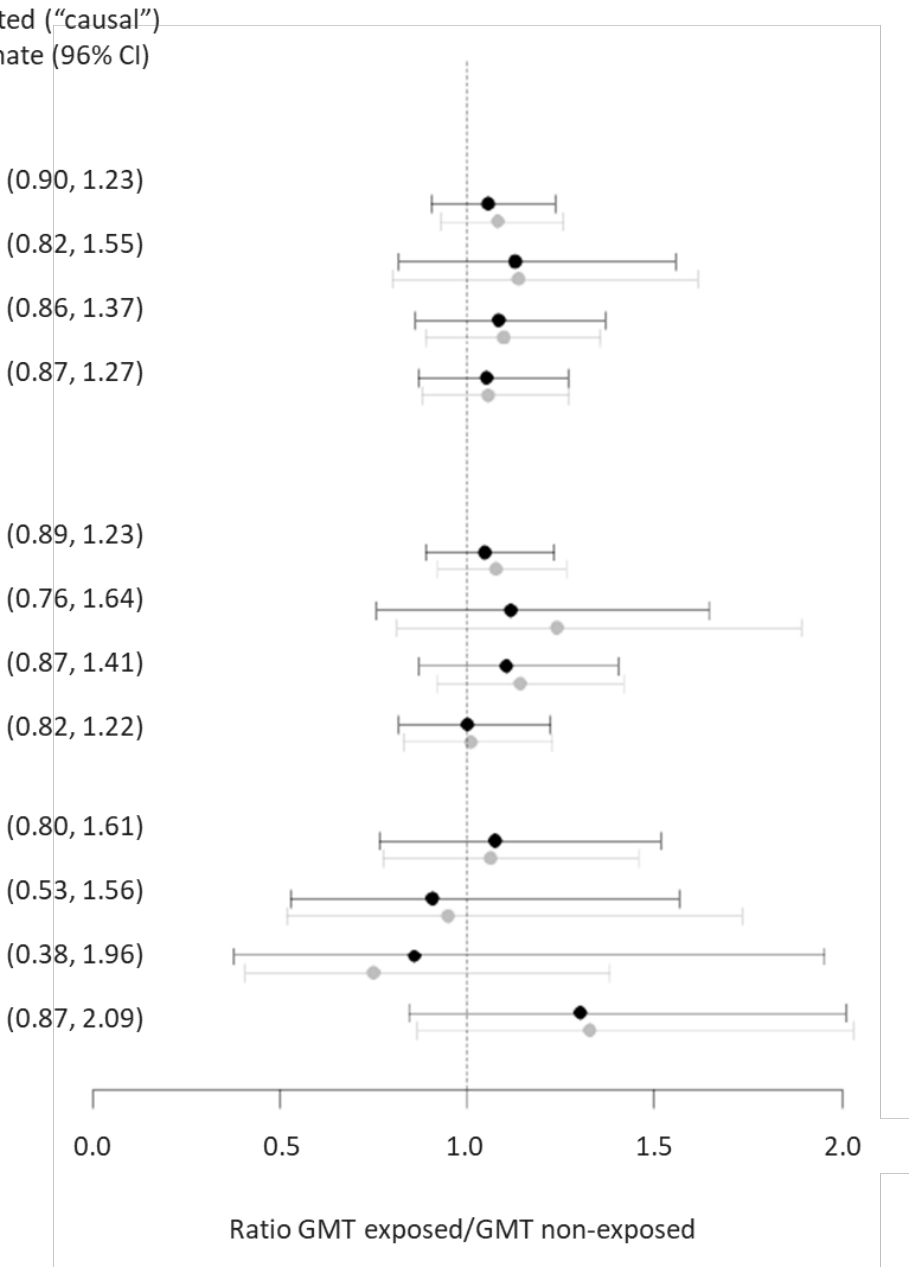

Unadjusted (“naive") estimates (grey) are based on a simple comparison of the observed responses in exposed and unexposed groups and are only reported for descriptive purposes. This estimate is expected to be biased as it does not potential confounders take into account. Adjusted (“causal”) estimates (black) are corrected for baseline imbalance in demographic factors and baseline characteristics between the exposed and control groups using inverse probability weighting. The weights were obtained from logistic regression modeling using the SuperLearner ensemble methodology. For analyses with ≥200 exposed participants, predictions were obtained using cross-validation; for analyses with <200 exposed participants within-sample predictions were used. The control group included 441 participants.

GMT, geometric mean titer; VNA, virus neutralization assay.

Figure S10. Fold-rise in vaccine-induced immune responses post RSV vaccination.

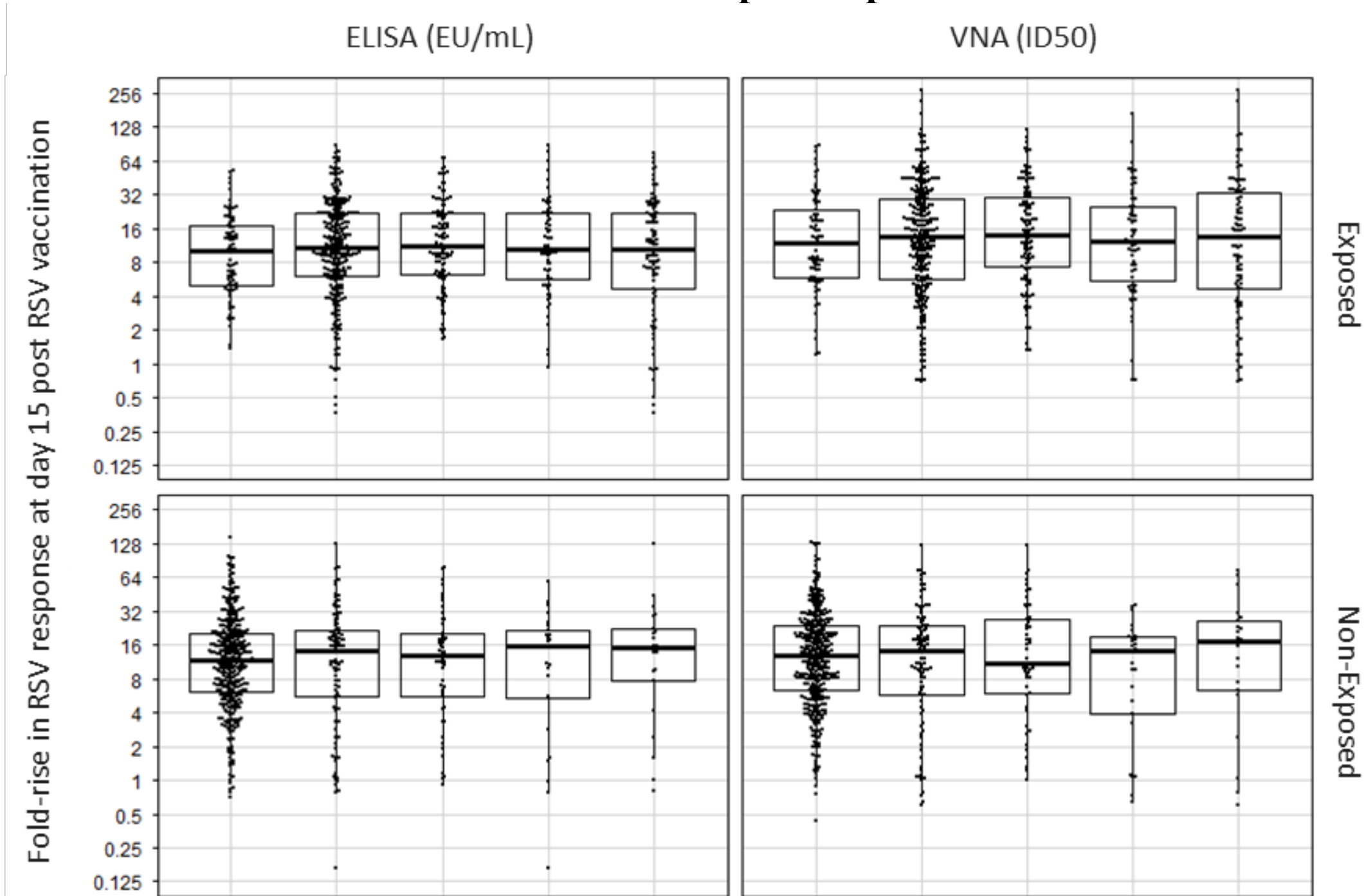

Ad26 NAb titer (IC90) At baseline		Negative	Positive	Low	Moderate	High	Negative	Positive	Low	Moderate	High
**Exposed**	N	78	270	113	64	93	78	270	113	64	93
	Median	10	11	11	10	10	12	13	14	12	13
	Interquartilerange	5-17	6-22	6-22	6-22	5-22	6-23	6-29	7-30	6-25	5-34
**Non-exposed**	N	343	98	50	26	22	343	98	50	26	22
	Median	12	14	13	16	15	13	14	11	14	17
	Interquartilerange	6-21	6-22	6-20	5-21	8-22	6-24	6-24	6-27	4-19	7-26

Boxplots presenting the median (50th percentile) fold-increase from baseline in RSV response measured by ELISA and VNA at Day 15 post RSV vaccination (Ad26.RSV.preF/protein) for participants who received a Ad26.COV2.S vaccination 1 to 12 months prior (exposed) and participants who did not previously receive any adeno-based vaccination (non-exposed) with boxes representing 25th percentile to 75th percentile. Whiskers extend to the lowest and highest value that is within 1.5 times the interquartile range. The individual participants datapoints are plotted over the boxplot as jittered dots. The corresponding table provides an overview of the total participants (N) and the median response and interquartile ranges. Participants were seronegative if the Ad26 NAb titer (IC_{90}) was <LLOD and seropositive if the Ad26 NAb titer (IC_{90}) was ≥LLOD. The magnitude of positive Ad26 NAb titers as demarcated into 3 ranges: ≤200 (low), >200 to ≤1,000 (moderate), and >1,000 (high).

ELISA, enzyme-linked immunosorbent assay; LLOD, lower limit of detection; NAb, neutralizing antibody; VNA, virus neutralization assay.

References

1 Sarzotti-Kelsoe, M. *et al.* Optimization and validation of the TZM-bl assay for standardized assessments of neutralizing antibodies against HIV-1. *J Immunol Methods* **409**, 131-146, doi:10.1016/j.jim.2013.11.022 (2014).

2 Milligan, I. D. *et al.* Safety and immunogenicity of novel adenovirus type 26- and modified vaccinia Ankara-vectored Ebola vaccines: a randomized clinical trial. *Jama* **315**, 1610-1623, doi:10.1001/jama.2016.4218 (2016).

3 Goldstein, N. *et al.* Safety and immunogenicity of heterologous and homologous 2-dose regimens of adenovirus serotype 26- and modified vaccinia Ankara-vectored Ebola vaccines: a randomized, controlled phase 1 study. *J Infect Dis* **226**, 595-607, doi:10.1093/infdis/jiaa586 (2022).

4 Mutua, G. *et al.* Safety and immunogenicity of a 2-dose heterologous vaccine regimen with Ad26.ZEBOV and MVA-BN-Filo Ebola vaccines: 12-month data from a phase 1 randomized clinical trial in Nairobi, Kenya. *J Infect Dis* **220**, 57-67, doi:10.1093/infdis/jiz071 (2019).

5 Anywaine, Z. *et al.* Safety and immunogenicity of a 2-dose heterologous vaccination regimen with Ad26.ZEBOV and MVA-BN-Filo ebola vaccines: 12-month data from a phase 1 randomized clinical trial in Uganda and Tanzania. *J Infect Dis* **220**, 46-56, doi:10.1093/infdis/jiz070 (2019).

6 Anywaine, Z. *et al.* Safety and immunogenicity of 2-dose heterologous Ad26.ZEBOV, MVA-BN-Filo Ebola vaccination in children and adolescents in Africa: a randomised, placebo-controlled, multicentre phase II clinical trial. *PLoS Med* **19**, e1003865, doi:10.1371/journal.pmed.1003865 (2022).

7 Barry, H. *et al.* Safety and immunogenicity of 2-dose heterologous Ad26.ZEBOV, MVA-BN-Filo Ebola vaccination in healthy and HIV-infected adults: a randomised, placebo-controlled phase II clinical trial in Africa. *PLoS Med* **18**, e1003813, doi:10.1371/journal.pmed.1003813 (2021).

8 Afolabi, M. O. *et al.* Safety and immunogenicity of the two-dose heterologous Ad26.ZEBOV and MVA-BN-Filo Ebola vaccine regimen in children in Sierra Leone: a randomised, double-blind, controlled trial. *Lancet Infect Dis* **22**, 110-122, doi:10.1016/S1473-3099(21)00128-6 (2022).

9 Ishola, D. *et al.* Safety and long-term immunogenicity of the two-dose heterologous Ad26.ZEBOV and MVA-BN-Filo Ebola vaccine regimen in adults in Sierra Leone: a combined open-label, non-randomised stage 1, and a randomised, double-blind, controlled stage 2 trial. *Lancet Infect Dis* **22**, 97-109, doi:10.1016/S1473-3099(21)00125-0 (2022).

10 Bockstal, V. *et al.* First-in-human study to evaluate safety, tolerability, and immunogenicity of heterologous regimens using the multivalent filovirus vaccines Ad26.Filo and MVA-BN-Filo administered in different sequences and schedules: A randomized, controlled study. *PLoS One* **17**, e0274906, doi:10.1371/journal.pone.0274906 (2022).

11 Barouch, D. H. *et al.* Evaluation of a mosaic HIV-1 vaccine in a multicentre, randomised, double-blind, placebo-controlled, phase 1/2a clinical trial (APPROACH) and in rhesus

monkeys (NHP 13-19). *Lancet* **392**, 232-243, doi:10.1016/S0140-6736(18)31364-3 (2018).
12 Stephenson, K. E. *et al.* Comparison of shortened mosaic HIV-1 vaccine schedules: a randomised, double-blind, placebo-controlled phase 1 trial (IPCAVD010/HPX1002) and a preclinical study in rhesus monkeys (NHP 17-22). *Lancet HIV* **7**, e410-e421, doi:10.1016/S2352-3018(20)30001-1 (2020).
13 Stieh, D. J. *et al.* Safety and immunogenicity of Ad26-vectored HIV vaccine with mosaic immunogens and a novel mosaic envelope protein in HIV-uninfected adults: a phase 1/2a study. *J Infect Dis* **227**, 939-950, doi:10.1093/infdis/jiac445 (2023).
14 Baden, L. R. *et al.* Safety and immunogenicity of two heterologous HIV vaccine regimens in healthy, HIV-uninfected adults (TRAVERSE): a randomised, parallel-group, placebo-controlled, double-blind, phase 1/2a study. *Lancet HIV* **7**, e688-e698, doi:10.1016/S2352-3018(20)30229-0 (2020).
15 Williams, K. *et al.* Phase 1 Safety and Immunogenicity Study of a Respiratory Syncytial Virus Vaccine With an Adenovirus 26 Vector Encoding Prefusion F (Ad26.RSV.preF) in Adults Aged >/=60 Years. *J Infect Dis* **222**, 979-988, doi:10.1093/infdis/jiaa193 (2020).
16 Comeaux, C. A. *et al.* Safety, Immunogenicity, and Regimen Selection of Ad26.RSV.preF-Based Vaccine Combinations: A Randomized, Double-blind, Placebo-Controlled, Phase 1/2a Study. *J Infect Dis* **229**, 19-29, doi:10.1093/infdis/jiad220 (2024).
17 Falsey, A. R. *et al.* Efficacy and safety of an Ad26.RSV.preF-RSV preF protein vaccine in older adults. *N Engl J Med* **388**, 609-620, doi:10.1056/NEJMoa2207566 (2023).
18 Sadoff, J. *et al.* Prevention of respiratory syncytial virus infection in healthy adults by a single immunization of Ad26.RSV.preF in a human challenge study. *J Infect Dis* **226**, 396-406, doi:10.1093/infdis/jiab003 (2022).
19 Sadoff, J. *et al.* Safety and immunogenicity of the Ad26.RSV.preF investigational vaccine coadministered with an influenza vaccine in older adults. *J Infect Dis* **223**, 699-708, doi:10.1093/infdis/jiaa409 (2021).
20 Stuart, A. S. V. *et al.* Phase 1/2a safety and immunogenicity of an adenovirus 26 vector Respiratory Syncytial Virus (RSV) vaccine encoding prefusion F in adults 18-50 years and RSV-Seropositive children 12-24 months. *J Infect Dis* **227**, 71-82, doi:10.1093/infdis/jiac407 (2022).
